# Enhancing functional antibody responses against HIV envelope V1V2 through vaccine formulations

**DOI:** 10.3389/fimmu.2025.1722596

**Published:** 2025-12-08

**Authors:** Clauvis Kunkeng Yengo, Xiaomei Liu, Gabriel Laghlali, Seok-Chan Park, Jéromine Klingler, Christina C. Luo, Xunqing Jiang, Xiang-Peng Kong, Priyanka G. Rao, Chitra Upadhyay, Matthew J. Wiest, Hiromi Muramatsu, Bruno G. De Geest, Pamela T. Wong, Ying Tam, Norbert Pardi, Michael Schotsaert, Catarina E. Hioe

**Affiliations:** 1 James J. Peters VA Medical Center, Bronx, NY, United States; 2 Division of Infectious Diseases, Department of Medicine, Icahn School of Medicine at Mount Sinai, New York, NY, United States; 3 Department of Microbiology, Department of Medicine, Icahn School of Medicine at Mount Sinai, New York, NY, United States; 4 Global Health and Emerging Pathogens Institute, Icahn School of Medicine at Mount Sinai, New York, NY, United States; 5 Department of Pharmaceutics, Ghent University, Ghent, Belgium; 6 Department of Biochemistry and Molecular Pharmacology, New York University Grossman School of Medicine, New York, NY, United States; 7 Department of Internal Medicine, University of Michigan Medical School, Ann Arbor, MI, United States; 8 Michigan Nanotechnology Institute for Medicine and Biological Sciences, University of Michigan Medical School, Ann Arbor, MI, United States; 9 Department of Microbiology, Perelman School of Medicine, University of Pennsylvania, Philadelphia, PA, United States; 10 Department of Biomedical Engineering, University of Michigan Medical School, Ann Arbor, MI, United States; 11 Acuitas Therapeutics, Vancouver, BC, Canada; 12 Icahn Genomics Institute, Icahn School of Medicine at Mount Sinai, New York, NY, United States; 13 Marc and Jennifer Lipschultz Precision Immunology Institute, Icahn School of Medicine at Mount Sinai, New York, NY, United States

**Keywords:** HIV, vaccine, V1V2, adjuvant, antibody, Fc function

## Abstract

**Background:**

Despite decades of research, the development of an effective HIV vaccine remains a significant challenge. Recent findings from three large vaccine efficacy trials have identified antibodies against the V1V2 domain of the HIV envelope glycoprotein as a potential correlate of reduced infection risk, offering a promising avenue for improving vaccine efficacy. Vaccine-elicited anti-V1V2 antibodies do not mediate potent virus-neutralizing activities, but they mediate Fc-dependent effector functions.

**Methods:**

This study evaluated the capacity of V1V2-scaffold vaccines in different formulations to generate antibody responses with Fc-mediated functions. BALB/c mice were immunized with V1V2-scaffold proteins formulated with one of the following adjuvants: MF59-like squalene-based oil-in-water emulsion (Addavax), a combination of TLR7/8 and RIG-I agonists (IMDQ-PC/IVT), nanoemulsion and RIG-I agonist (NE/IVT), or empty lipid nanoparticles (eLNP). All formulations were administered intramuscularly except NE/IVT, which was given intranasally. For comparison, we also tested a V1V2-scaffold-expressing mRNA-LNP vaccine delivered intramuscularly and an Env gp140 protein with liposomal MPLA/DDA adjuvant administered subcutaneously.

**Results:**

Among the six vaccine formulations tested, V1V2-scaffold immunogens adjuvanted with LNP (eLNP and mRNA-LNP) elicited the most robust and cross-reactive serum IgG responses that recognized native Env on cell surfaces or virions. The eLNP and mRNA-LNP groups, along with IMDQ-PC/IVT, also elicited functional IgG2a, and correspondingly displayed Fc-mediated activities, as measured by antibody-dependent cellular phagocytosis and FcγRIV binding. Notably, IMDQ-PC/IVT elicited predominantly IgG2a with minimal IgG1, eLNP stimulated IgG1 and IgG2a with IgG1 dominance, whereas mRNA-LNP yielded more balanced IgG2a/IgG1 responses.

**Conclusions:**

Data from this study provide new insights into the utility of novel formulations for V1V2-scaffold immunogens as a strategy for optimizing the induction of functional V1V2-specific antibodies to improve HIV vaccine efficacy.

## Introduction

Over 40 years after the discovery of HIV, an effective HIV vaccine is still unavailable. Data from three Phase 2b/3 vaccine trials identified V1V2-specific antibodies as a signal correlated with reduced infection risk. The Thai RV144 trial, which tested ALVAC-HIV with bivalent AIDSVAX B/E gp120 in alum, demonstrated 60% efficacy at one year and 31.2% by year 3.5 (1, 2) and revealed a correlation between high levels of anti-V1V2 IgG and lower infection risk (3–7). Although broadly neutralizing antibodies were not induced, antibodies mediating Fc effector functions, including IgG3, were detected (4, 5, 7–9), and Fc functions such as antibody-dependent cellular cytotoxicity (ADCC) and complement deposition (ADC’D) inversely correlated with infection risk in a subset of vaccine recipients (3, 4, 9). The HVTN 702 trial in South Africa, which adapted the RV144 vaccine regimen with sub-Saharan African C/C gp120 antigens and MF59 adjuvant, failed to show efficacy (10). Yet, case-control analyses found that an interaction of polyfunctional CD4 T cell responses and high V1V2 IgG levels correlated with reduced HIV risk (11). Similarly, HVTN 705 (Imbokodo), testing mosaic Ad26 and gp140 vaccines, presented no efficacy and low V1V2-specific antibody responses, though higher V1V2 IgG3 breadth scores trended toward decreased infection risk across multiple statistic models (12).

Non-human primates (NHP) studies confirmed the association between strong anti-V1V2 antibody responses and reduced susceptibility to SIV or SHIV challenge (13–22). Passive transfer studies further revealed Fc-mediated protective effects of non-neutralizing anti-Env monoclonal antibodies (mAbs). For example, AAV-delivered non-neutralizing 5L7 IgG1 mAb showed protective effects linked to ADCC against repeated SIVmac239 challenge (23). Non-neutralizing V2-specific mAb NCI05 also delayed SIVmac251 acquisition in correlation with mucosal NCI05 levels and CD14 cell-mediated efferocytosis (24). Collectively, these studies suggest that vaccines capable of eliciting high-level durable V1V2-specific antibodies with potent Fc activity could provide meaningful protection, but developing such vaccines remains a significant challenge.

Fc-mediated effector activities depend on antibody-antigen complexes engaging Fc receptors (FcRs) or complement (25, 26). These interactions are largely dictated by antibody isotypes and subclasses (27–32). In mice, of the four IgG subclasses, IgG1 has low affinities for FcγR and complement, whereas IgG2a/c binds FcγRI and FcγRIV strongly and recruits C1q efficiently (33–35). IgG2b binds FcγRIV and C1q with moderate affinity, while IgG3 binds C1q and not FcγRs. In humans, IgG1 and IgG3 interact with FcRs and C1q effectively, while IgG2a and IgG4 have weak or no binding (36, 37).

Adjuvants influence not only magnitude of antibody responses but also isotype/subclass distribution, thereby shaping Fc effector functions against various antigens, including HIV Env (38–40), influenza (41–43), and MERS spike (44). Of note, an adjuvant combination consisting of a modified TLR7/8 agonist (imidazoquinoline-PEG-ChoI [IMDQ-PC]) and RIG-I agonist (*in vitro* transcribed Sendai virus defective-interfering RNA [IVT]) used with inactivated influenza virus vaccine elicited predominantly IgG2a with minimal IgG1, correlating with greater ADCC against influenza HA and NA (42). TLR7/8 agonist alone also shifted responses to IgG2a but retained some IgG1. IgG2a skewing by TLR7/8 agonist was reiterated with SARS-CoV-2 spike vaccine, which conferred protection against virus challenge (45). In contrast, RIG-I agonist alone maintained IgG1 dominance, similar to that observed with research-grade MF59-equivalent adjuvant, Addavax (42). An intranasal adjuvant made of IVT in nanoemulsion (NE/IVT) augmented serum IgG1, IgG2b, and IgG2c responses to SARS-CoV-2 receptor binding domain (RBD) as well as IgG and IgA levels in bronchoalveolar lavage fluid (46). Ionizable lipid nanoparticles (LNP), used widely to encapsulate mRNA vaccines, also display potent adjuvant activity, as indicated by the ability of empty LNPs (eLNP) to augment the immunogenicity of influenza HA and SARS-CoV-2 spike proteins, eliciting IgG2a, IgG2b, and IgG1 (47). Nonetheless, these different adjuvants have not been extensively evaluated with the inherently less immunogenic HIV vaccines.

HIV Env-based vaccines, including soluble gp120, membrane-bound Env, trimeric soluble gp140 SOSIP or UFO, elicit strong responses to immunogenic sites but inconsistently induce V1V2-specific antibodies (48–56). In RV144, one-third of participants generated high-level anti-V1V2 antibodies, while in HVTN 702, only 3% did (11). To improve this, we previously designed structure-guided immunogens presenting V1V2 on heterologous scaffolds. Among nine scaffolds presenting six V1V2 strains tested in rabbits (57, 58) and NHPs (59, 60), AE.A244 V1V2-scaffold constructs delivered as protein and DNA vaccines emerged as most immunogenic, eliciting V1V2 Abs with Fc functionality (59, 61). However, optimization of V1V2 vaccine formulations to enhance Fc-mediated functional responses remains largely unexplored.

This study compared V1V2-scaffold vaccine formulations for the ability to elicit Fc-functional antibody responses in mice. V1V2-scaffold protein immunogens were formulated with Addavax (squalene-based oil-in-water emulsion), IMDQ-PC/IVT (TLR7/8 and RIG-I agonists) (42), NE/IVT (nanoemulsion and RIG-I agonist) (46), and eLNP (47). We also tested a V1V2-scaffold expressing mRNA vaccine (mRNA-LNP)−encapsulated in LNP incorporating the same ionizable lipid as the eLNP (47)−and a UFO Env trimeric protein (56). The data showed that these formulations elicited V1V2 IgG responses of varying magnitude and breadth across HIV strains/clades, with recognition of multiple Env formats including soluble gp120 and gp140 Env proteins as well as membrane-bound Env on cells and virions. Analysis of Ig isotypes/subclasses revealed differential IgG1 vs. IgG2a profiles that correlated with Fc functional potency. Notably, LNP-adjuvanted V1V2 vaccines (protein with eLNP and mRNA-LNP) induced high-level cross-clade V1V2 IgG responses marked by IgG2a and Fc functionality, while IMDQ-PC/IVT uniquely promoted IgG2a-dominant responses with minimal IgG1.

## Materials and methods

### Vaccine preparation

V1V2-scaffold protein immunogens, AE.A244 V1V2-2J9C and AE.A244 V1V2-1FD6, were expressed and purified according to a published protocol (58) with some modifications. Plasmids encoding the respective constructs were transfected into HEK293S GnTI^-^ cells using the JetPRo^®^ transfection reagent (Polyplus) and cultured in FreeStyle™ 293 expression medium (Gibco) at 37°C with 8% CO_2_ on a rotating platform (110–130 rpm). Culture supernatants were harvested 72–120 hours post-transfection and clarified by centrifugation (3,000 rpm, 10 minutes). V1V2-scaffold proteins were purified using Ni-nitrilotriacetic acid (Ni-NTA) agarose beads, eluted with 600 mM imidazole, desalted using spin desalting columns (ThermoFisher), and then concentrated with 10 kDa Amicon^®^ Ultra centrifugal filters (Sigma). Endotoxin was removed using the Triton X-114 phase separation method as previously described (62) to achieve <0.01 EU/mL residual concentration as measured with the ToxinSensor™ Endotoxin Detection System (GenScript). Each protein preparation was sterile-filtered, analyzed for purity and size by 12% SDS-PAGE, and stored in aliquots at -80°C until use. Antigenicity was verified by ELISA using a panel of human monoclonal antibodies against V1V2 and other Env regions.

**Figure 1:**
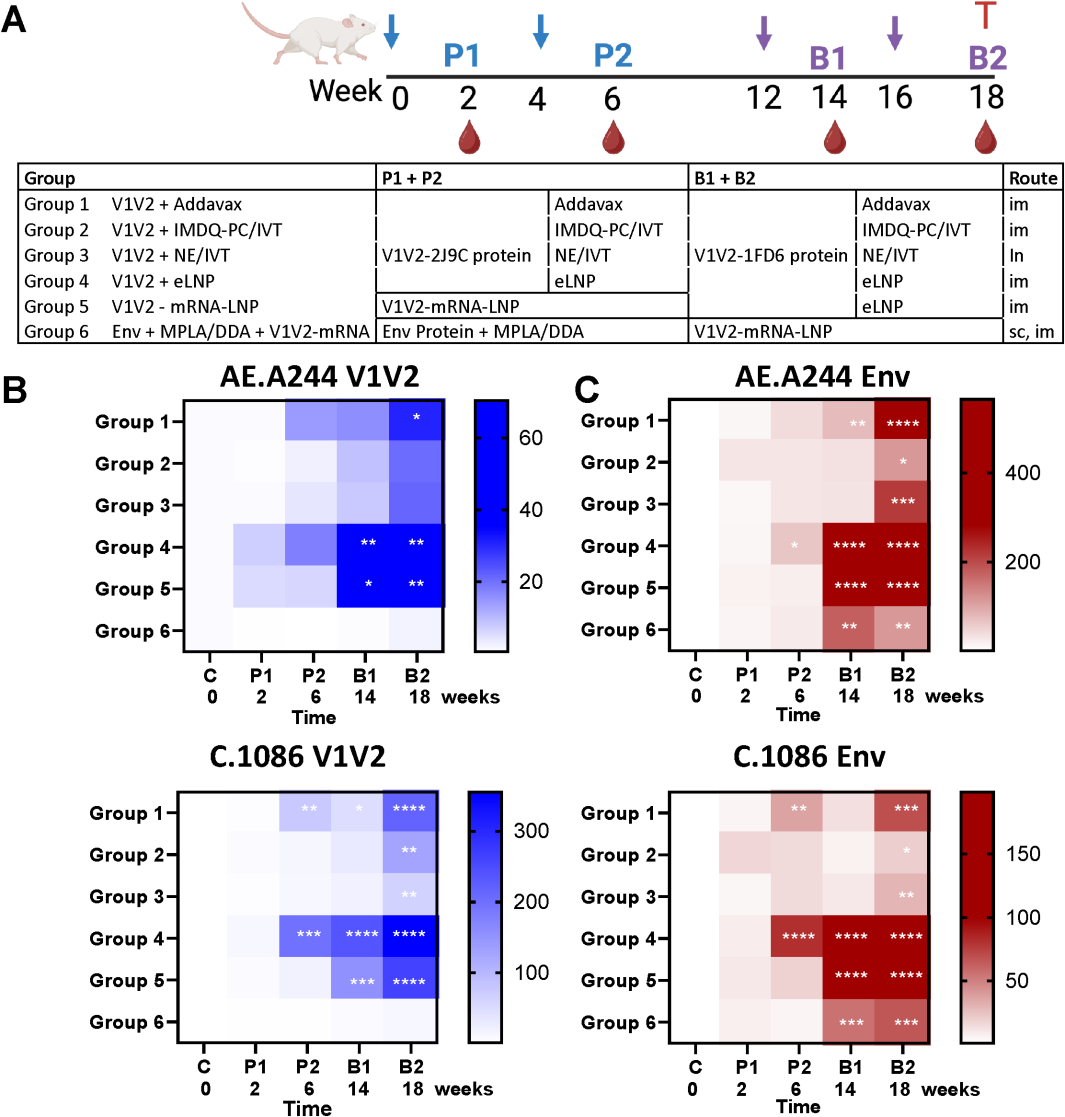
Serum IgG responses against V1V2 and Env antigens from homologous AE.A244 and heterologous C.1086 strains following immunization with V1V2 and Env immunogens in various formulations. **(A)** Schematic of the immunization protocol. Six groups of BALB/c mice (n=5 per group) were immunized with V1V2 or Env vaccines derived from the AE.A244 strain, administered in different formats and adjuvants via intramuscular (im), intranasal (in), or subcutaneous (sc) routes. Each group received two priming doses followed by two booster doses at designated time points. Created with BioRender.com. **(B, C)** Serum IgG responses to V1V2 **(B)** and Env **(C)** antigens of the homologous AE.A244 and heterologous C.1086 strains. Serum samples were collected after each immunization and analyzed at a 1:60 dilution using a Luminex bead-based multiplex assay to quantify antigen-specific IgG responses. Results are presented as median fold increases over pre-bleed controls and visualized using heat maps. Statistical significance was assessed using Kruskal-Wallis with Dunn’s multiple comparisons test comparing post-vaccination IgG levels to pre-bleed controls within each group. *p < 0.05; **p < 0.01; ***p < 0.001; ****p < 0.0001; unmarked p ≥ 0.05.

On the day of immunization, V1V2-scaffold protein (3 μg/dose) was combined with the adjuvants tested in the study: i) AddaVax™ (InvivoGen, 1:1 (vol/vol) ratio) (42), ii) IMDQ-PC/IVT consisting of IMDQ-PEG-Chol (130 μg, equivalent to 10 μg core IMDQ, per dose) co-formulated with IVT RNA (1 μg SDI-RNA with 40 μg nanogel per dose) (42), iii) NE/IVT prepared with 20% NE and 0.5 μg of IVT RNA in a 12 µL dose (63), and iv) empty LNP (eLNP, 300 μg lipid/dose). In addition, AE.A244 V1V2-2J9C-expressing mRNA-LNP (10 μg mRNA/dose) was thawed and diluted in PBS. eLNP (eLNP) and mRNA-LNP were prepared as previously described (47, 64); the ionizable lipid and LNP composition are described in the international patent application WO2017075531(2017). The mRNA-LNP were 79 nm with a polydispersity index (PDI) <0.025 and an encapsulation efficiency of >95%; for the eLNP, particles were 64 nm with a PDI 0.043. A bivalent gp140 UFO Env protein immunogen (3 µg/dose total, consisting of a 1:1 mixture of AE.A244 and A.BG505 UFO trimers was adjuvanted with monophosphoryl lipid A (MPL-A, 25 µg/dose; Avanti) and dimethyldioctadecylammonium (DDA, 250 µg/dose; Sigma) (56). The UFO proteins were made in the laboratory of Dr. Xiang-Peng Kong (New York University) as reported previously (56). All vaccines were diluted with sterile PBS to deliver 50 µL/dose/animal for intramuscular or subcutaneous immunizations, except for NE/IVT (12 µL/dose) for intranasal immunization, and stored on ice until administration. The same doses were administered for priming and boosting vaccines according to the schematics in **Figure 1A**.

### Immunization and sample collection

All animal procedures were reviewed and approved by the Institutional Animal Care and Use Committee (IACUC) of the Icahn School of Medicine at Mount Sinai and conducted in accordance with institutional ethical guidelines. Female BALB/c mice (6–8 weeks old) were purchased from the Jackson Laboratory and housed in a specific pathogen-free facility at the Icahn School of Medicine at Mount Sinai. Mice were randomly assigned to six groups (n=5/group). Each group received vaccination via a designated route of administration at time intervals outlined in **Figure 1A**. Blood was collected from submandibular vein two weeks after each injection. At the end of the study, vaginal washes were obtained with 25 μL PBS per animal, and terminal blood was collected by cardiac puncture. Specimens were heat-inactivated at 56°C for 30 minutes prior to use.

### Multiplex bead antibody-binding assay

Mouse IgG reactivity to autologous and heterologous Env and V1V2 antigens was quantified using Luminex multiplex bead assays using a panel of antigens that included gp140, gp120, and V1V2-tags proteins previously described (56, 57, 60). IgG binding to B.REJO/c and B.JRFL IMC virions was measured using a protocol adapted from (65), in which sucrose cushion-purified pre-titrated virions were covalently coupled to Luminex beads with the xMAP antibody coupling kit. Diluted mouse sera or vaginal washes were incubated with the beads, and bound antibodies were detected using biotinylated anti-mouse IgG (Rockland Biotech), IgG1, IgG2a, or IgA (Southern Biotech) antibodies followed by PE-labeled streptavidin (Abacam). Data were acquired on a Luminex Bio-Plex FlexMAP 3D system, and antigen-specific reactivity was reported as median fluorescence intensity (MFI) or fold increase over negative control.

Recombinant HIV-1 proteins AE.A244 Δ11 gp120 (ARP-12569), C.1086 gp140C (ARP-12581), C.1086 gp140C K160N (ARP-12580), C.1086 gp120 D7 (ARP-12582), M.CON-S Δ11 gp120 (ARP-12576), C.1086 V1V2 tags, and AE.A244 V1V2 tags were obtained through the NIH HIV Reagent Program, Division of AIDS, NIAID, NIH, and were contributed by Drs. Barton F. Haynes and Hua-Xin Liao. Recombinant gp120 JRFL was purchased from Immune Technology.

### Cell-associated Env binding assay

Antibody binding to cell surface-expressed HIV Env was evaluated using a previously described protocol (66, 67) using 293T cells transfected with Env gp160-expressing plasmid (Polyplus jetPEI). After 24-hour transfection, cells were treated with Live/Dead Fixable Aqua viability dye and then incubated with serially diluted mouse sera for 30 minutes on ice. IgG binding was detected using Alexa 647-labeled goat anti-mouse IgG (1:1000, Invitrogen) on live cells with an Attune NxT flow cytometer. IgG binding levels across serum dilutions were quantified as percentage of positive cells.

### Assays to measure Fc-mediated activities

Antibody-dependent cellular phagocytosis (ADCP) activity mediated by V1V2-specific antibodies in mouse sera was evaluated as previously described (48, 56, 68) using fluorescent NeutrAvidin beads conjugated with V1V2-tags antigens and THP-1 phagocytic cells (American Type Culture Collection (ATCC). ADCP scores were calculated across serum dilutions.

To measure FcγRIV binding, recombinant His_6_-tagged FcγRIV protein (10 µg/mL, R&D Systems) was used. The FcγRIV binding to antibody-antigen complexes on Luminex beads were detected using anti-His-PE antibody (R&D Systems) according to a protocol adapted from (69).

### Neutralization assay

Neutralization activity of mouse sera was evaluated using a standardized HIV pseudovirus assay with TZM.bl reporter cells (56, 70). Neutralization titers were expressed as the serum dilution at which relative luminescence units (RLUs) were reduced by 50% (ID_50_), as compared to untreated virus wells (set to 0%) and cell only wells (set to 100%). PG9 monoclonal antibody produced in the Hioe lab was used as positive control. TH023.06 WT or N160K Env-expressing plasmids were gifts from Drs. David Montefiori and Shaunna Shen (Duke University). The following reagent was obtained through the NIH HIV Reagent Program, Division of AIDS, NIAID, NIH: Human Immunodeficiency Virus Type 1 (HIV-1) SG3ΔEnv Non-infectious Molecular Clone, ARP-11051, contributed by Dr. John C. Kappes and Dr. Xiaoyun Wu.”

### Statistical analysis

All data analyses were performed using GraphPad Prism (GraphPad Software).

## Results

### Prime-boost immunization with V1V2-scaffold immunogens to target the V1V2 region of HIV Env

To elicit V1V2-focused antibody responses, we tested two V1V2-scaffold proteins (V1V2-2J9C and V1V2-1FD6 (56–58, 61, 71)) with different adjuvants in a prime-boost vaccination regimen (**Figure 1A**). Both V1V2-scaffolds were constructed to present V1V2 of an HIV-1 CRF01_AE strain A244 (AE.A244). Distinct scaffolds were utilized to promote V1V2 targeting while averting anti-scaffold responses. BALB/c mice (n=5/group) received two priming doses of V1V2-2J9C protein on weeks 0 and 4 and two boosting doses of V1V2-1FD6 protein on weeks 12 and 16. Four adjuvants were tested: Addavax (Group 1), IMDQ-PC/IVT (Group 2), NE/IVT (Group 3), and eLNP (Group 4). Additionally, mice were primed with two doses of nucleoside-modified mRNA-LNP expressing V1V2-2J9C and boosted with two doses of V1V2-1FD6 protein adjuvanted with eLNP (Group 5). For comparison, we also immunized mice with two doses of an uncleaved prefusion optimized (UFO) Env trimer composed of AE.A244 gp120 and A.BG505 gp41 in MPLA/DDA adjuvant, followed with two V1V2-2J9C mRNA-LNP boosts (Group 6). Primes and boosts were administered via an intramuscular route to ipsilateral thighs, except for V1V2 in NE/IVT, which was designed for intranasal delivery (Group 3), and UFO Env in MPLA/DDA, which was injected subcutaneously to ipsilateral flanks (Group 6). Blood samples were collected two weeks after each vaccine dose, and vaginal washes were acquired after the last vaccination.

### Robust cross-reactive serum IgG responses elicited by LNP-adjuvanted V1V2-scaffold mRNA and protein immunogens

Longitudinal serum samples were analyzed for the kinetics of antibody generation against V1V2-tags and Env (gp120 or gp140) of homologous AE.A244 and heterologous C.1086 strains that lacked the 2J9C and 1FD6 scaffold components to allow detection of antibodies that specifically recognize V1V2 on its own and in the context of Env. IgG responses were detected above control against homologous AE.A244 V1V2 and heterologous C.1086 V1V2 after the second priming doses (P2) in Groups 1-5 (**Figure 1B**, **Supplementary Figure S1**), and the elicited IgG were reactive with AE.A244 Env and C.1086 Env (**Figure 1C**, **Supplementary Figure S1**). Following two boosting doses (B1 and B2), IgG levels against V1V2 and Env of both AE.A244 and C.1086 increased further, albeit to varying extents among groups (**Figures 1B, C**). Groups 4 and 5 showed the greatest IgG responses against V1V2 and Env of AE.A244 and C.1086.

Animals in Group 6 generated minimal IgG responses against V1V2 of AE.A244 and C.1086 after Env priming (**Figure 1B**), although responses to Env were readily detectable above background (**Figure 1C**), indicating poor V1V2 immunogenicity on Env. Anti-V1V2 IgG responses were generated only after boosting with V1V2-2J9C mRNA-LNP, although the magnitudes were relatively weak (**Figures 1B, C**; **Supplementary Figure S1**).

**Figure 2:**
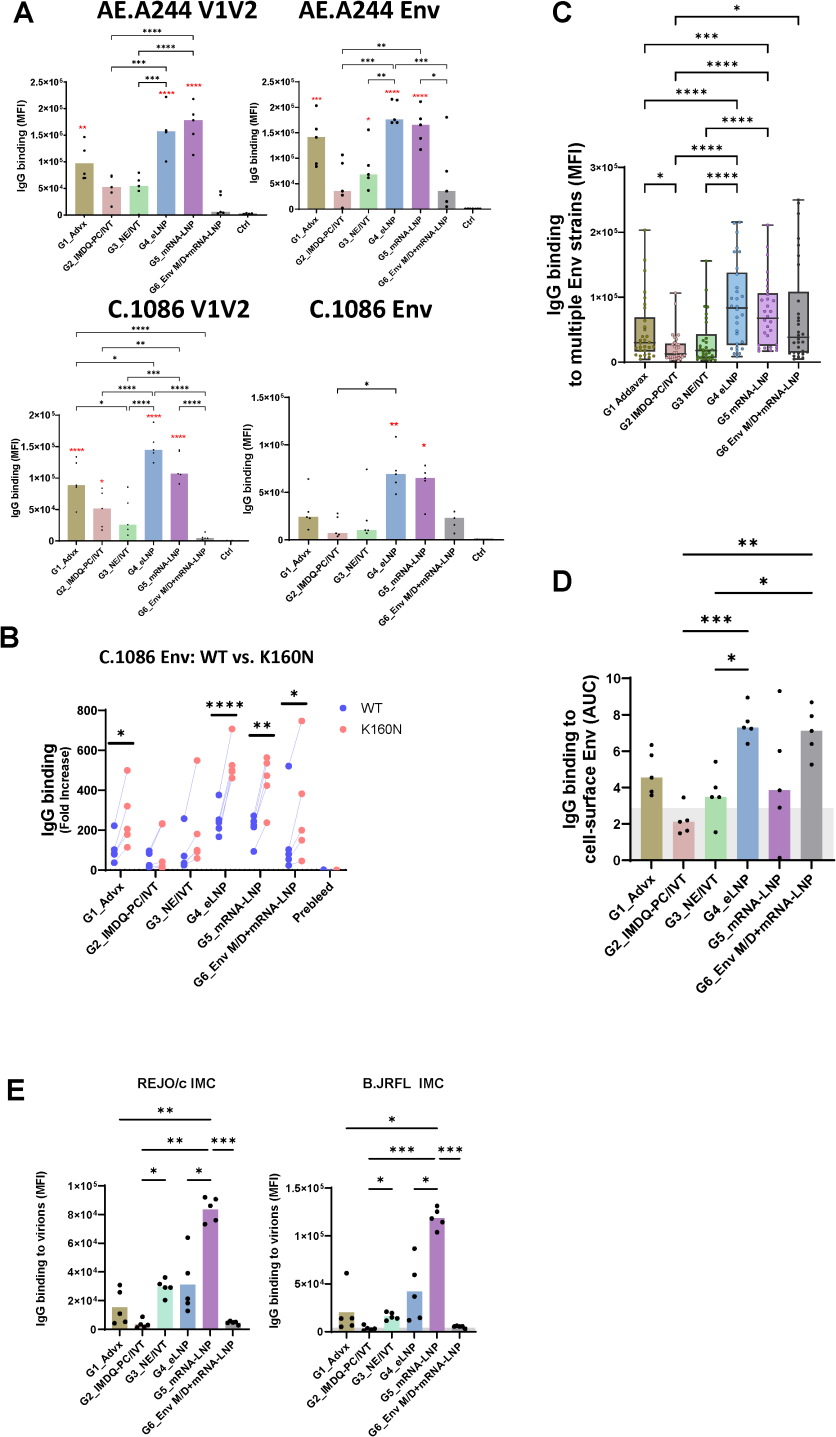
The magnitude and breadth of vaccine-induced serum IgG responses after the final vaccination. **(A)** Levels of serum IgG to V1V2 and Env antigens from AE.A244 and C.1086 were assessed two weeks after the final immunization across six vaccine groups. Prebleed samples served as baseline controls. Antibody binding was measured using a multiplex bead-based assay, and the results are presented as median fluorescence intensity (MFI). Statistical comparisons were conducted using one-way ANOVA with Tukey’s multiple comparison test. Red asterisks in panel A indicate significance compared to control (ctrl); black asterisks indicate significance between immunized groups. **(B)** To assess the targeting of V1V2 apical surface, IgG recognition of trimeric C.1086 Env wild type (WT) or K160N mutant was compared. The K160N substitution introduces an N160 glycosite on the V1V2 apex. Statistics were done using paired t test. **(C)** Breadth of IgG responses was evaluated based on binding to Env proteins from multiple HIV-1 strains and subtypes: AE.A244 gp120 and gp140, C.1086 gp140 and gp120, M.Cons gp120 and B.JRFL gp120. Statistical comparison was performed using one-way ANOVA with Tukey’s multiple comparison test. **(D)** IgG reactivity to cell surface Env was measured by flow cytometry using 293T cells transfected to express AE.92TH03.06 Env. Serially diluted individual sera were incubated with the cells and IgG binding was detected with a AF647-labeled anti-mouse IgG secondary antibody. Statistical analysis was performed using one-way ANOVA with Tukey’s multiple comparison test. Gray area: negative control. **(E)** IgG reactivity with HIV-1 virions was examined using virion-coupled Luminex beads. Individual serum samples (diluted 1:60) were incubated with the beads and IgG binding was detected with fluorescence-labeled secondary antibodies specific for mouse IgG. Statistical analysis was performed using one-way ANOVA with Tukey’s multiple comparison test. Gray area: negative control. ****p<0.0001; ***p<0.001; **p<0.01; *p<0.05; p≥0.05 values are left unmarked.

Comparison of IgG responses elicited after the final vaccination (B2) showed that high magnitudes of IgG against V1V2 and Env of AE.A244 and C.1086 were elicited in Groups 4 and 5, as well as in Group 1 to lower extents (**Figure 2A**). Of note, both groups 4 and 5 were boosted with V1V2-1FD6 in eLNP adjuvant. Group 4 had also the most elevated V1V2 and Env-specific IgG responses after two priming doses (P2) with V1V2-2J9C in eLNP (**Figures 1B, C**; **Supplementary Figure S2A**), indicating the superior adjuvanticity of eLNP.

To assess the targeting of V1V2 apex, we measured the relative levels of IgG binding to C.1086 gp140 Env wild type or a mutant (K160N) with a N160 glycosylation site in the V1V2 apex. Increased IgG reactivity to the mutant over wild type was detected across all six groups after two boosts (B2) (**Figure 2B**) and, to smaller extents, after two priming doses (P2) (**Supplementary Figure S2B**).

The breadth of IgG reactivity was further assessed across six Env antigens from different HIV-1 subtypes (AE.A244 gp120 and gp140, C.1086 gp140 and gp120, M.Cons gp120 and B.JRFL gp120) using individual sera collected after the final immunization. The data showed that Group 4 displayed the greatest IgG cross-reactivity, followed by Group 5 (**Figure 2C**, **Supplementary Figure S3A**). These groups received LNP-adjuvanted V1V2 protein and/or mRNA-LNP vaccines. Analysis of IgG reactivity to only heterologous Env antigens yielded a similar pattern (**Supplementary Figure S3B**). We also evaluated IgG recognition of heterologous Env (AE.92TH023.06) expressed on the cell surface and observed variable reactivity across groups, with Group 4 showing the highest binding and Group 2 the lowest (**Figure 2D**). Group 6 also exhibited strong cell-surface Env binding, most likely directed to epitopes outside V1V2. Furthermore, we measured IgG binding to HIV virions and a distinct pattern emerged: Group 5 showed the highest level of IgG reactivity with virions, while Groups 2 and 6 had no detectable binding (**Figure 2E**). This pattern was similar for the two HIV-1 infectious molecular clones (IMC) tested, B.REJO/c (transmitted/founder, T/F) and B.JRFL (chronic). Altogether, these results demonstrated the superior capacity of LNP as an adjuvant for V1V2-scaffold protein and mRNA vaccines to elicit robust serum IgG responses against diverse HIV strains. However, IgG binding across Env formats varied substantially, reflecting differences in Env recognition that depend on the antigen source.

### Induction of vaginal IgA following intranasal vaccination with V1V2-scaffold protein vaccines in NE/IVT adjuvant

In addition to circulating antibodies, mucosal antibodies at the site of virus entry also can influence HIV transmission through the mucosal routes. To evaluate whether antibodies were elicited in the mucosa following intranasal immunization with V1V2-scaffold proteins in NE/IVT (Group 3), as compared to systemic immunization with the other adjuvants, we measured IgA and IgG in vaginal washes collected from individual mice after the last vaccination (**Supplementary Figures S4**-**S5**). Weak, albeit significant, levels of AE.A244 Env-binding IgA were detected above control in vaginal washes (diluted 1:30) from mice vaccinated with V1V2-scaffolds in NE/IVT (Group 3) and not in the other five groups (**Supplementary Figure S4A**). However, no IgA reactivity was measurable above control against C.1086 Env, AE.A244 V1V2, and C.1086 V1V2 antigens. Env- and V1V2-specific IgG antibodies were also detected at near or below background in these vaginal washes (**Supplementary Figure S5A**).

In contrast, strong vaginal IgA responses were detected in Group 3 against the scaffold 2J9C and 1FD6 proteins incorporated in the V1V2 immunogens, as evidenced by high IgA levels against all V1V2-2J9C and V1V2-1FD6 antigens tested, irrespective of the V1V2 strains (**Supplementary Figure S4B** and data not shown). The other five groups showed no or sporadically low IgA responses to the scaffolds. In addition, vaginal IgG responses to the scaffolds were induced in Groups 1-5, although the levels did not rise significantly above background (**Supplementary Figure S5B** and data not shown). These data demonstrated the utility of NE/IVT as an intranasal adjuvant for mounting IgA and IgG responses in the vaginal mucosa, albeit it was insufficient for bolstering the responses to V1V2.

### Elicitation of serum neutralizing activity and Fc-mediated effector functions across six vaccine groups

**Figure 3:**
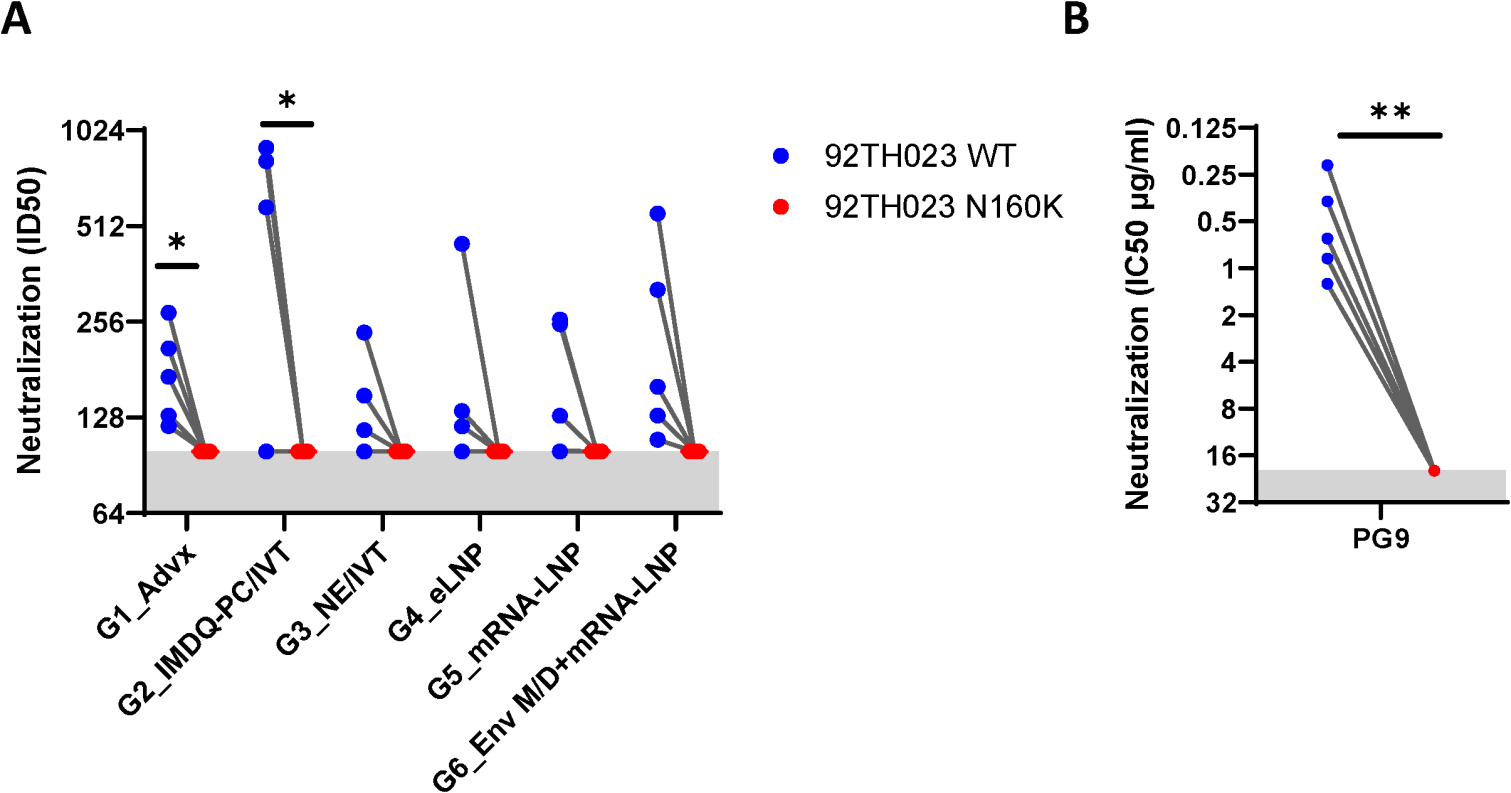
Virus neutralization in sera from immunized mice from six vaccine groups. Serially diluted serum samples from the final time point (B2) were tested for neutralization of HIV-1 pseudoviruses expressing Env of TH023.06 WT or N160K in a standard assay using TZM.bl target cells. Neutralization titers are reported as serum dilution required to inhibit 50% of virus infection (ID50) **(A)** Serum from unimmunized mice served as a negative control (ID50 <100, gray area). PG9 (V2 apex bNAb) sensitive to N160K mutation at the V2 apex was tested as a positive control in each plate alongside mouse sera **(B)** *p<0.05; **p<0.01 by paired t test.

Vaccination regimens incorporating V1V2-scaffolds have been shown to elicit serum antibodies with low neutralizing activity against sensitive tier 1 viruses and a few more resistant tier 2 viruses. To examine whether serum antibodies elicited in the six vaccine groups had neutralizing activity, serially diluted individual samples from the final time point were evaluated against tier 1 AE.92TH023.06 pseudovirus in the standard neutralization assay using TZM.bl reporter cells. An N160K mutant lacking the N160 glycosite at the V1V2 apex was also tested in parallel to determine V1V2 targeting. Varying neutralization titers were detected above background in sera from all or some of the animals in each of the six vaccine groups, and no significant differences were observed across the groups (**Figure 3A**). While overall the ID50 titers were low, the activities were uniformly abrogated by the N160K mutation, indicating N160-dependent virus neutralization, similar to that seen with the V2 glycan-dependent bNAb PG9 (**Figure 3B**).

**Figure 4:**
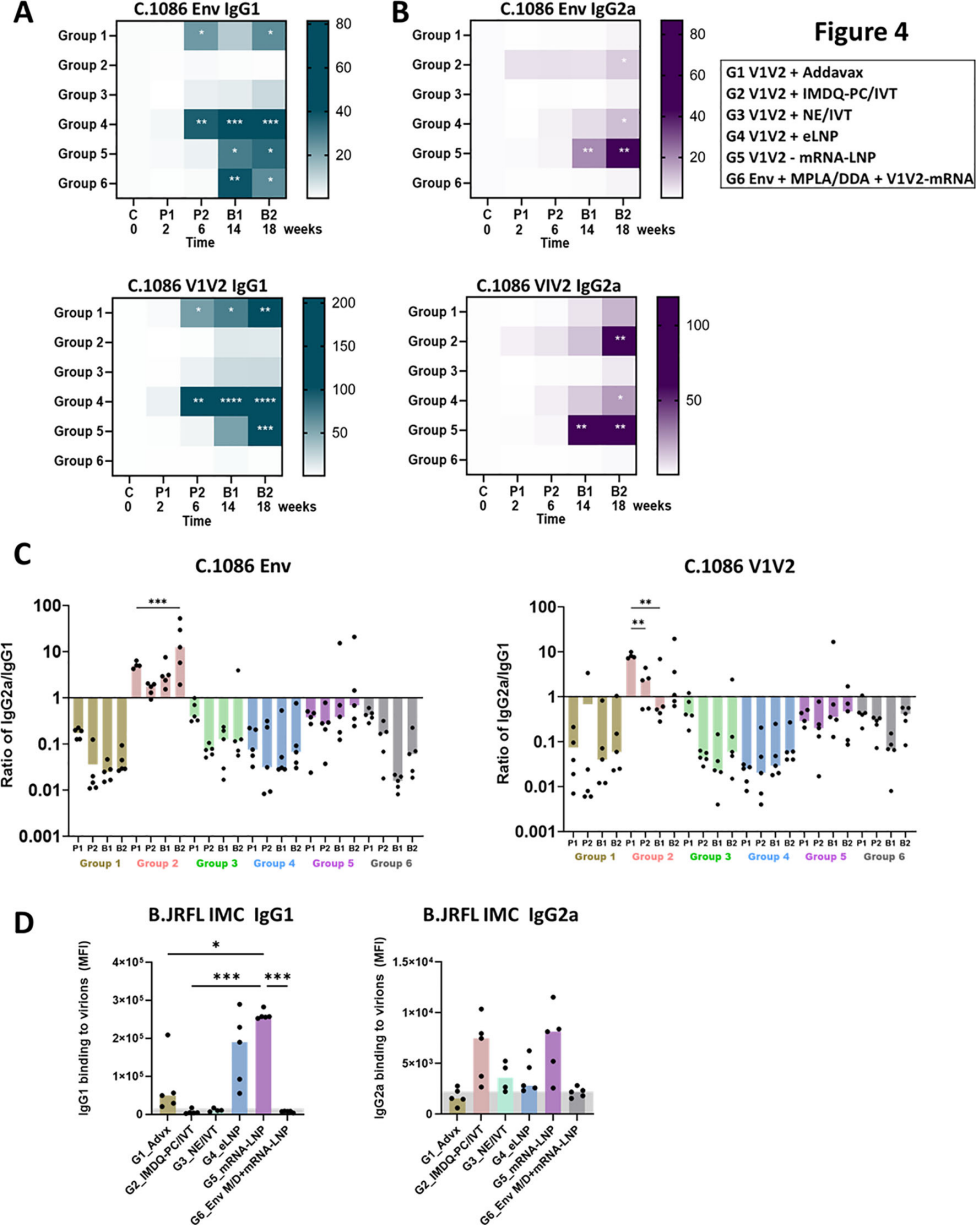
Serum IgG1 and IgG2a responses against V1V2 and Env antigens from heterologous antigens in six vaccine groups. **(A, B)** Serum IgG1 **(A)** and IgG2a **(B)** responses to C.1086 Env and V1V2 antigens. Serum samples collected after each immunization were analyzed at a 1:60 dilution using a Luminex bead-based multiplex assay to quantify antigen-specific IgG1 vs. IgG2a responses. Results are presented as median fold increases over pre-bleed controls and visualized using heat maps. Statistical significance was assessed using Kruskal-Wallis with Dunn’s multiple comparisons test comparing post-vaccination IgG levels to pre-bleed controls within each group. **(C)** Ratio of IgG2a/IgG1 responses to C.1086 Env and VIV2 antigens of the heterologous C.1086 **(C)** strains. Statistical differences between post-vaccination time points for each group was assessed by one-way ANOVA with Sidak’s multiple comparison test. **(D)** IgG1 and IgG2a reactivity with B.JRFL IMC virions coupled on Luminex beads. Statistical test was done to compare vaccine groups by one-way ANOVA with multiple comparison test. *p < 0.05; **p < 0.01; ***p < 0.001; ****p < 0.0001; unmarked p ≥ 0.05.

In addition to virus neutralization, Fc-mediated effector functions of antibodies also contribute to anti-viral responses. Because Fc activities are dictated by the isotypes and subtypes of the antibodies, we evaluated the levels of IgG1 and IgG2a that represent two murine IgG subtypes with poor and strong Fc functional potential, as well as indicators of Th2 and Th1 responses, respectively (72, 73). Serum samples collected two weeks after each vaccination were tested for IgG1 and IgG2a reactivity against C.1086 Env and V1V2. IgG1 responses increased progressively with each vaccine dose, except for Group 2, which received V1V2-scaffold immunogens adjuvanted in IMDQ-PC/IVT and showed IgG1 levels near background (**Figures 4A, B**). The highest IgG1 levels were attained in Group 4. In contrast, IgG2a responses were detected prominently, with high variability across animals, in Group 2 (V1V2 + IMDQ-PC/IVT), Group 4 (V1V2 + eLNP), and Group 5 (V1V2-mRNA-LNP and V1V2 + eLNP), consistent with the ability of these adjuvants to stimulate Th1 responses (45, 47). Similar profiles were observed for the reactivity to AE.A244 Env (**Supplementary Figures S6A, B**). Of note, the bias toward IgG2a versus IgG1 was most pronounced for Group 2 (V1V2 + IMDQ-PC/IVT), yielding IgG2a/IgG1 ratios of >1 in this group only (**Figure 4C**, **Supplementary Figure S6C**), in line with past reports showing the special property of this adjuvant in the context of other vaccines (42). Group 4 induced IgG2a and IgG1 but IgG1 remained dominant. Group 5 generated IgG2a/IgG1 ratios closer to 1, indicating more balanced IgG2a/IgG1 responses. After the last vaccine dose, Groups 2 and 5 also displayed IgG2a reactivity with B.JRFL IMC, whereas virus-binding IgG1 was detected in Groups 4 and 5 and at lower extents Group 1 (**Figure 4D**).

In a separate study, we examined the immunogenicity of AE.A244 gp120 protein when formulated in Addavax (i.m.), IMDQ-PC/IVT (i.m.) or NE/IVT (i.n.) adjuvants vs. no adjuvant. In contrast to V1V2-scaffold immunogens, gp120 protein adjuvanted in IMDQ-PC/IVT elicited weak or no IgG2a responses against homologous and heterologous Env, and the IgG2a/IgG1 ratios did not increase above 1, indicating lack of IgG2a skewing (**Supplementary Figure S7**). Immunization with gp120 in either Addavax or NE/IVT elicited predominantly IgG1 responses (**Supplementary Figure S7**), similar to those seen with V1V2-scaffold immunogens (**Figure 4**, **Supplementary Figure S6**).

To evaluate Fc effector functions, two different assays were used: i) a flow-cytometry-based ADCP assay using THP-1 effector cells and antigen-coated fluorescent beads and ii) a Luminex-based Fc receptor binding assay using antigen-coated beads and recombinant FcγRIV protein. Serum samples from the last time point (after four vaccine doses) were tested in both assays.

**Figure 5:**
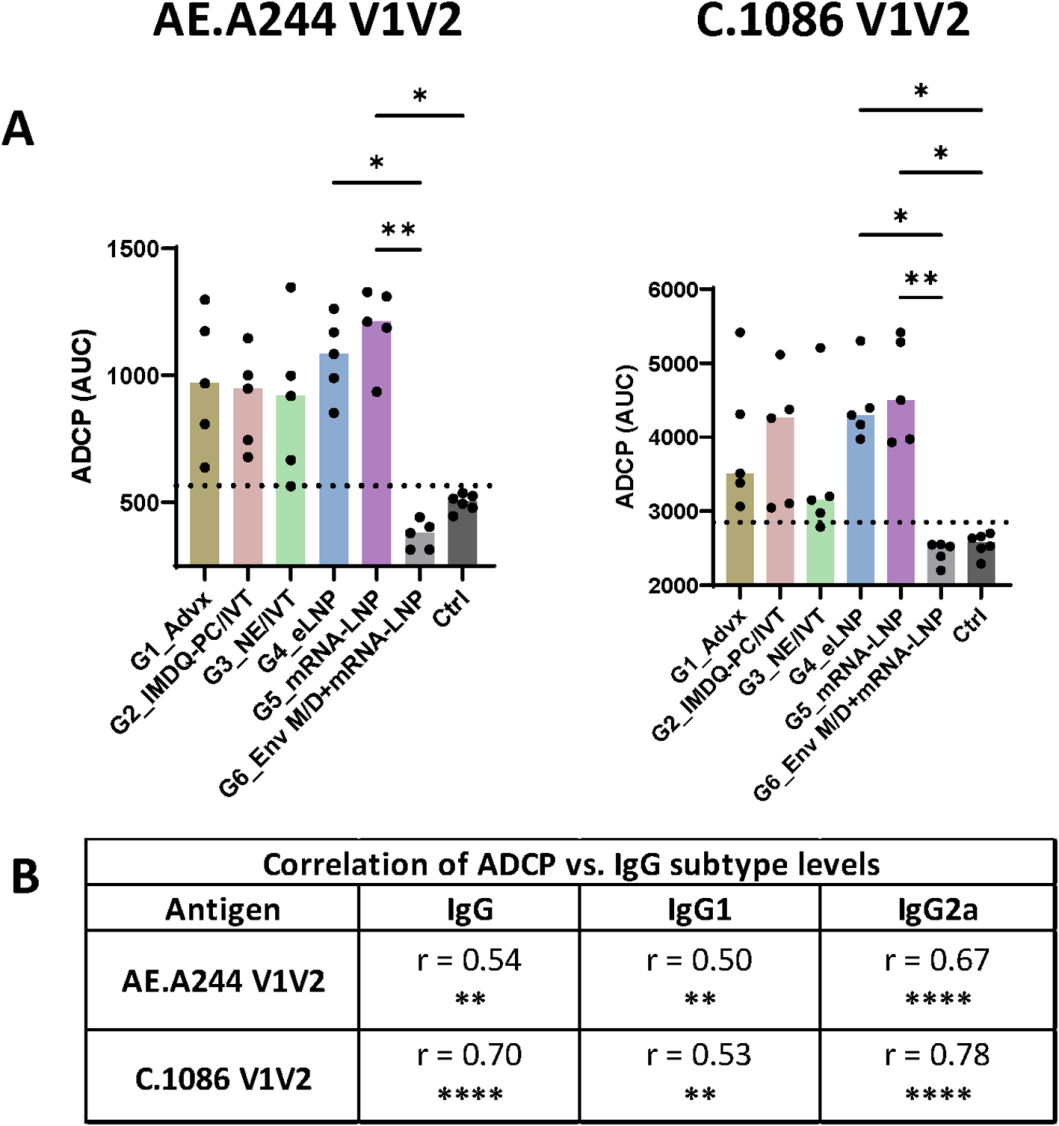
V1V2-specific ADCP activity across six vaccine groups. **(A)** The ability of V1V2-specific antibodies to mediate ADCP was measured using a flow cytometry-based assay with the THP-1 phagocytic cell line. Serially diluted samples from the final time point were incubated with beads coated with antigens and then with THP-1 cells. Titration curves for individual samples are shown in **Supplementary Figure S8**. The area under the curves (AUC) was calculated for each sample and is presented here. Pooled pre-bleed samples tested in six replicates were used as negative controls (Ctrl). Dotted line: mean+2SD of controls. Statistical comparison among groups was performed using one-way ANOVA with Kruskal-Wallis multiple comparison test. *p<0.05, **p<0.01, p≥0.05 (no mark). **(B)** Spearman correlation analyses were performed to evaluate the association between ADCP and total IgG, IgG1, and IgG2a levels for each antigen. Correlation coefficient (r) and p values are shown. **p<0.01, ****p<0.0001, ns (not significant) p≥0.05.

Varying levels of ADCP activity against V1V2 of AE.A244 and C.1086 were detected in Groups 1-5, but not in Group 6 (Env+MPLA/DDA priming and V1V2-mRNA-LNP boosts) (**Figure 5A**, **Supplementary Figure S8**). The highest ADCP levels were displayed by Group 5 followed by Group 4 and Group 2, with variability observed across animals especially in Group 2. Groups 1 and 3 also displayed highly variable levels of V1V2-specific ADCP. The ADCP magnitudes correlated most strongly with IgG2a levels, followed with IgG and IgG1 levels (**Figure 5B**), confirming the key contribution of IgG2a relative to IgG1 to Fc effector activities.

**Figure 6:**
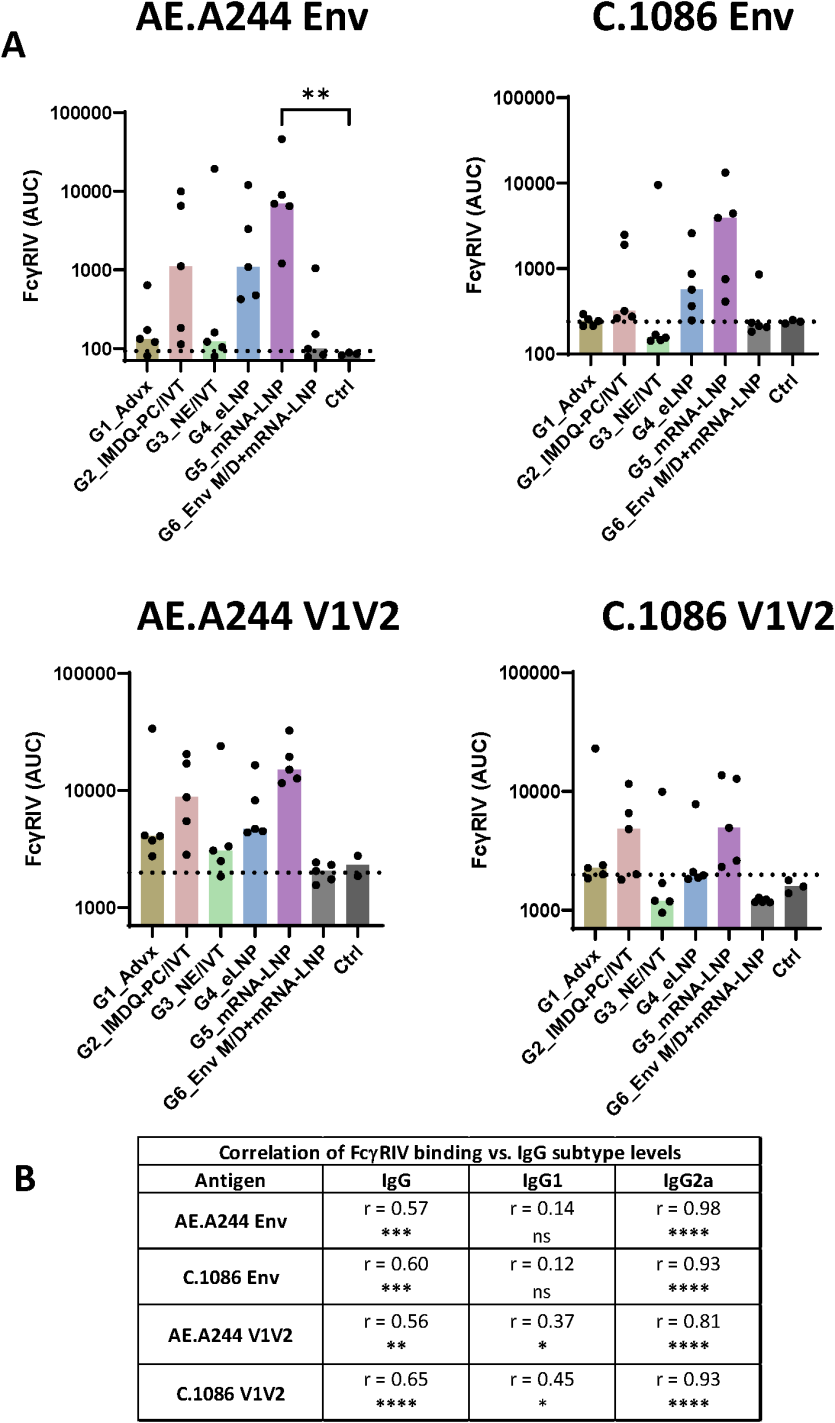
FcγR binding by Env and V1V2-specific serum antibodies across six vaccine groups. **(A)** The ability of Env- and V1V2-specific antibodies to engage FcγRIV was measured using a Luminex bead-based assay. Serum samples collected at the final time point were titrated and incubated with beads coated with AE.A244 Env, C.1086 Env, AE.A244 V1V2, or C.1086 V1V2 antigens. Recombinant His_6_-tagged FcγRIV protein (10 µg/mL) was added, and binding to antibody-antigen complexes was detected using anti-His tag antibodies. Titration curves for individual samples are shown in **Supplementary Figure S9**. The area under the curves (AUC) was calculated for each sample and is presented here. Pre-bleed samples were used as negative controls (Ctrl). Dotted line: mean+2SD of controls. Statistical comparison among groups was performed using one-way ANOVA with Kruskal-Wallis multiple comparison test. **p<0.01, p≥0.05 (no mark). **(B)** Spearman correlation analyses were performed to evaluate the association between FcγRIV binding and total IgG, IgG1, and IgG2a levels for each antigen. Correlation coefficient (r) and p values are shown. * p<0.05, ** p<0.01, ***p <0.001, ****p<0.0001, ns (not significant) ≥0.05.

Vaccine-induced antibodies from mice in Group 5, Group 4, and Group 2 also displayed FcγRIV binding (**Figure 6A**, **Supplementary Figure S9**). Again, the binding levels varied greatly among individual animals in each group and across the four antigens tested, and a significant increase above control was observed only for FcγRIV binding by AE.A244 Env-specific antibodies from Group 5. In contrast, Groups 1, 3, and 6 had low or no FcγRIV binding activity. We also noted that no animals in Group 6 had detectable FcγRIV binding activity to V1V2-specific antibodies, similar to that seen with ADCP. Like ADCP, FcγRIV binding levels correlated most strongly with IgG2a, followed by total IgG and IgG1 (**Figure 6B**). Altogether, these data provided evidence for the capacity of LNP- and IMDQ-PC/IVT-adjuvanted V1V2-scaffold vaccines to elicit IgG2a antibodies with potent Fc effector functions, albeit with distinct profiles. IMDQ-PC/IVT induced predominantly IgG2a responses at relatively low magnitudes, eLNP generated both IgG1 and IgG2a while maintaining IgG1 dominance, and mRNA-LNP mounted more balanced IgG2a and IgG1 responses.

## Discussion

This study demonstrated the critical contribution of vaccine adjuvants and formulations in shaping both the magnitude and quality of antibody responses to V1V2 immunogens. By testing different adjuvants with the same V1V2 scaffold proteins, we observed varying levels of V1V2-specific IgG responses elicited in immunized mice. Among the tested adjuvants, eLNP (Group 4) displayed clear superiority over Addavax (Group 1), IMDQ-PC/IVT (Group 2), and NE/IVT (Group 3), inducing higher and broader serum IgG responses against V1V2 and Env antigens, including cell surface-expressed Env. The increase in IgG levels was particularly pronounced after V1V2-1FD6 boosts following V1V2-2J9C priming, revealing for the first time the advantages of using two heterologous scaffolds to present V1V2 for prime-boost vaccination. We also examined V1V2-2J9C mRNA-LNP vaccination (Group 5), which stimulated low antibody responses against V1V2 and Env, but were markedly enhanced by boosting with V1V2-1FD6 adjuvanted with eLNP, thereby recapitulating the potent adjuvanticity of eLNP. In contrast, priming with the UFO Env vaccine in MPLA/DDA (Group 6), an adjuvant used in several prior studies (48, 56), generated Env-specific responses that did not target V1V2. Boosting this group with V1V2-2J9C mRNA-LNP induced IgG responses to V1V2, albeit at levels considerably lower than in other groups. Whether stronger anti-V1V2 responses can be attained by boosting with eLNP-adjuvanted V1V2-1FD6 protein, as in Group 5 remains to be determined. Together, these findings support the notion that the poor immunogenicity of V1V2 in the context of Env could be overcome by engrafting V1V2 onto exogenous scaffolds. However, defining the optimal prime-boost regimens that incorporate different V1V2-scaffold immunogens will require more systematic comparative studies.

The modulatory effects of vaccine adjuvants on IgG subtype distribution were evident in the context of V1V2-scaffold vaccination. Consistent with prior influenza vaccine studies (42), Addavax (Group 1) elicited predominantly IgG1 responses with limited Fc potency. In contrast, IMDQ-PC/IVT (Group 2) induced a unique profile characterized by Fc-functional IgG2a and negligible IgG1, although IgG2a responses were relatively low and variable across animals. Immunization with V1V2-scaffold proteins using the intranasal adjuvant NE/IVT (Group 3) mounted only low-level IgG1 responses without detectable IgG2a. Vaginal IgA antibodies were also detected in this group; however, they were directed against the scaffolds rather than V1V2. Similarly, intranasal administration of an NE/IVT-adjuvanted gp120 protein vaccine failed to elicit IgG2a and IgA responses. These outcomes contrast with earlier reports in C57BL/6 mice, where intranasal immunization with SARS-CoV-2 spike protein in NE/IVT induced robust serum IgG1, IgG2b, and IgG2c responses with Th1 polarization (IgG2b/c bias), as well as bronchoalveolar lavage IgA antibodies (74, 75). Whether differences in mouse strain account for the divergent outcomes remains to be determined. Nevertheless, the robust antibody responses detected against the scaffold in sera and vaginal secretions, but not against V1V2 and gp120, point to an inherently weak immunogenicity of HIV Env. These data underscore the particular challenge of developing HIV vaccines capable of stimulating potent functional antibody responses to V1V2 and other vulnerable regions of Env.

In addition to IMDQ-PC/IVT, LNP-adjuvanted vaccines (protein + eLNP, Group 4; mRNA-LNP, Group 5) also induced IgG2a responses, although eLNP still showed IgG1 bias and mRNA-LNP yielded a more balanced IgG2a/IgG1 ratio. Correspondingly, antigen-specific Fc-mediated functions, such as ADCP and FcγR binding, were observed in all three groups producing IgG2a, with Fc activity magnitude correlating most strongly with IgG2a levels. We postulate that the vaccine-elicited IgG2a, rather than IgG1, may predict vaccine efficacy; however, this present study was limited to immunogenicity assessments and did not evaluate protective effects against HIV infection, which would require *in vivo* virus-challenge models. The mechanisms underlying the distinct adjuvant properties were also not addressed. Prior work has shown that IMDQ-PC/IVT, but not Addavax, upregulates acute ISG15 gene expression (42). Similarly, as compared to Addavax, eLNP and mRNA-LNP trigger higher early cytokine and chemokine expression, including IL-6 production critical for LNP adjuvant activity and for supporting germinal center B cell and follicular helper T cell responses (47). Nonetheless, systematic comparisons of innate responses induced across adjuvants remain limited. Future experiments are needed to determine whether each formulation elicits a distinct innate immune signature that imprints the quality of the ensuing adaptive immune responses, including Ig class switching.

We previously demonstrated that immunization of BALB/c mice with AE.A244 gp120 complexed with an anti-V2, anti-CD4bs, or anti-V3 monoclonal antibody enhanced the induction of V1V2-specific antibodies compared to the uncomplexed counterpart (48). However, the MPLA/DDA adjuvant was utilized and no V1V2-specific ADCP activity was observed (48), similar to the outcome in Group 6 (UFO Env+MPLA/DDA priming with V1V2-mRNA-LNP boosts). In another study, co-immunization of rabbits with AE.A244 V1V2-2J9C DNA vaccines and MPLA/DDA-adjuvanted AE.A244 UFO Env protein, delivered either as immune complex or uncomplexed protein, generated V1V2-specific antibodies capable of mediating ADCP, but these antibodies displayed limited cross-reactivity, primarily recognizing the homologous AE.A244 V1V2 and Env antigens (56). The present study showed that immunization with V1V2-scaffold immunogens bearing only the AE.A244 sequence generated antibody responses with cross-clade reactivity and the ability to recognize native Env on cells and virions. Nonetheless, the breadth remained limited, as denoted by low IgG reactivity to M.Cons and B.JRFL Env antigens, suggesting insufficient coverage across diverse HIV-1 strains and clades. Notably, utilizing a cocktail of V1V2-scaffolds presenting different V1V2 strains may provide a practical strategy to broaden antibody reactivity, as supported by previous immunogenicity data from rhesus macaques immunized with an array of V1V2-scaffold immunogens (59).

This study has additional limitations. The relatively small sample size and variability across animals within each group constrained our ability to fully assess statistical significance, particularly for comparison of IgG2a responses and Fc functions, which were nonetheless observed in three of the six vaccine groups. While ideally prophylactic HIV vaccines should elicit neutralizing and non-neutralizing antibodies capable of interrupting virus infection at the urogenital mucosa, the primary site of virus transmission through sexual contacts, the V1V2 vaccine formulations tested here, including the NE/IVT adjuvant designed specifically for mucosal delivery, elicited negligible vaginal IgG and IgA against V1V2 and Env. It should be noted, however, that vaginal or rectal delivery of this adjuvant had not yet been evaluated. Moreover, dose-titration studies were not performed, precluding the determination of optimal V1V2-scaffold doses for each adjuvant tested.

## Conclusion

V1V2-scaffold constructs were tested along with different adjuvants and formulations for the ability to elicit broadly reactive IgG antibodies with Fc functionality. Using two V1V2-scaffolds for a prime-boost approach, we demonstrated for the first time that V1V2-scaffold immunogens administered as protein mixed with IMDQ-PC/IVT, eLNP, or as mRNA-LNP were able to generate cross-reactive serum IgG2a responses capable of recognizing native Env on cells and virions and mediating Fc-dependent effector functions. Although further optimization is required, these findings provide a foundation for the rational selection of adjuvants and vaccine platforms in the development of V1V2-based vaccines and, more broadly, for vaccines designed to elicit antibodies with potent Fc-mediated functions.

## Data Availability

The raw data supporting the conclusions of this article will be made available by the authors, without undue reservation.

## Supplementary material

Supplementary Figure 1 Serum IgG responses against V1V2 and Env antigens from homologous AE.A244 **(A)** and heterologous C.1086 **(B)** strains in six vaccine groups. Serum samples were collected after each immunization and analyzed at a 1:60 dilution using a Luminex bead-based multiplex assay to quantify antigen-specific IgG responses. MFI: median fluorescence intensity. *p < 0.05; **p < 0.01; ***p < 0.001; ****p < 0.0001; unmarked p ≥ 0.05 vs. prebleed controls (dotted lines) by Kruskal-Wallis with Dunn’s multiple comparisons.

Supplementary Figure 2 Vaccine-induced serum IgG responses after two priming immunizations. **(A)** Total serum IgG binding to V1V2 and Env antigens from AE.A244 and C.1086 was measured two weeks after the second priming vaccination in six groups of mice. Statistical comparisons between groups were performed using one-way ANOVA with Tukey’s multiple comparison test. ****p<0.0001; ***p<0.001; **p<0.01; *p<0.05; no mark for p≥0.05. Red asterisks indicate comparisons to the prebleed controls (ctrl); black asterisks denote comparisons between immunized groups. **(B)** IgG recognition of trimeric C.1086 Env wild type (WT) or K160N mutant was compared to assess the effect of a K160N substitution that introduces an N160 glycosite on the V1V2 apex. Statistical analyses was performed with paired t test to compare reactivity to WT vs. K160N. *p<0.05; no mark for p≥0.05. MFI: median fluorescence intensity.

Supplementary Figure 3 Breadth of vaccine-induced IgG responses to Env antigens from different HIV-1 strains and subtypes. **(A)** Serum IgG reactive to heterologous and homologous Env proteins (gp120 or gp140) was assessed two weeks after the final immunization in six vaccine group. Individual serum samples were tested at a 1:60 dilution using a multiplex bead-based assay. Prebleed serum pool and bovine serum albumin (BSA) serve as background controls. **(B)** IgG breadth to only heterologous Env antigens were compared across six vaccine groups after the last immunization. Statistical comparison was performed using one-way repeated measured ANOVA with Tukey’s multiple comparison test. **p<0.01; p≥0.05 (no mark shown). MFI: median fluorescence intensity.

Supplementary Figure 4 Vaginal IgA responses to Env and V1V2 antigens in six vaccine groups. Vaginal wash samples were collected two weeks after the final immunization and assessed for IgA reactivity against Env and V1V2 antigens of the AE.A244 and C.1086 strains **(A)**. In addition, IgA responses to the V1V2-2J9C protein—a representative scaffold immunogen used in the vaccination regimen— were evaluated **(B)**. All samples were tested at a 1:30 dilution using a multiplex bead-based assay. Control samples were obtained from unvaccinated animals. Dotted line: cut-off (mean + 2SD of control). MFI: median fluorescence intensity. **p<0.01 by one-way ANOVA with multiple comparison test. Red asterisks indicate comparisons to the prebleed controls (ctrl); black asterisks denote comparisons between immunized groups.

Supplementary Figure 5 Vaginal IgG responses to Env and V1V2 antigens in six vaccine groups. Vaginal wash samples collected two weeks after the final immunization were assessed for IgG binding to Env and V1V2 of the AE.A244 and C.1086 strains **(A)**. IgG responses to the V1V2-2J9C antigen, included in the vaccination regimen, were also evaluated **(B)**. All samples were tested at a 1:30 dilution using a multiplex bead-based assay. Samples from unvaccinated animals served as negative controls. Statistical analyses using one-way ANOVA with multiple comparison test showed no significant difference versus control and across six vaccine groups (p ≥0.05, no mark). MFI: median fluorescence intensity. Dotted line: cut-off (mean + 2SD of control).

Supplementary Figure 6 Serum IgG1 and IgG2a responses against AE.A244 V1V2 and Env in six vaccine groups. (A–B) Serum IgG1 **(A)** and IgG2a **(B)** responses were measured following each immunization. Sera were analyzed at a 1:60 dilution using a Luminex bead-based multiplex assay. Results are presented as median fold increases over pre-bleed controls and visualized via heat maps. Statistical significance was determined using the Kruskal-Wallis test with Dunn’s multiple comparisons, assessing differences between post-vaccination and baseline IgG level within each group. **(C)** Ratio of IgG2a/IgG1 responses to Env and VIV2 antigens of the homologous AE.A244 strains. Statistical significance was assessed by one-way ANOVA with multiple comparison test. *p < 0.05; **p < 0.01; ***p < 0.001; ****p < 0.0001; unmarked p > 0.05.

Supplementary Figure 7 Serum IgG1 and IgG2a responses to Env after vaccination with AE.A244 gp120 protein formulated in different adjuvants. **(A, B)** Mice (n=5/group) received four doses of recombinant AE.A244 gp120 protein vaccines formulated in Addavax (i.m.), IMDQ-PC/IVT (i.m.), NE/IVT (i.n.) or no adjuvant. Mock control mice received PBS (i.m.) with no antigen or adjuvant (PBS_Ctrl). Two weeks after the fourth vaccination serum samples were collected and tested at 1:60 dilution using the Luminex bead-based multiplex assay to measure IgG1 and IgG2 responses to gp120 Env antigens of the autologous AE.A244 **(A)** or C.1086 **(B)** strains. Statistical significance was assessed using the Kruskal-Wallis test with Dunn’s multiple comparisons to determine differences among the five groups. *p < 0.05; **p < 0.01; unmarked p ≥ 0.05 (not significant). Dotted line: mean +2SD of PBS control. **(C)** IgG2a/IgG1 ratios were also calculated to assess IgG2a bias. Notably, gp120 formulated in IMDQ-PC/IVT adjuvant did not induce IgG2a-skewed responses, in contrast to responses observed with V1V2-scaffold proteins and the same adjuvant (**Figure 4**, **Supplementary Figure S6**).

Supplementary Figure 8 V1V2-specific ADCP activity of titrated serum antibodies across six vaccine groups. Serially diluted serum samples collected at the final time point from each vaccine group were incubated with V1V2-conjugated beads and THP-1 phagocytic cells. ADCP was quantified by flow cytometry and quantified as ADCP score (x10000). Pooled pre-bleed samples were used as negative control (orange lines).

Supplementary Figure 9 FcγR binding by titrated serum antibodies against AE.A244 Env, AE.A244 V1V2, C.1086 Env, and C.1086 V1V2 antigens across six vaccine groups. Serially diluted serum samples from each vaccine group were incubated with the indicated antigens. The binding of FcγR protein to the antibody-antigen complexes was quantified and are presented as median fluorescence intensity (MFI). Pooled pre-bleed samples were used as negative control (orange lines).
