# Supplementary figures and images for: Enhancing functional antibody responses against HIV envelope V1V2 through vaccine formulations
Source: Front Immunol. 2025 Dec 8;16:1722596. doi: 10.3389/fimmu.2025.1722596 (PMC12719441; doi:10.3389/fimmu.2025.1722596)

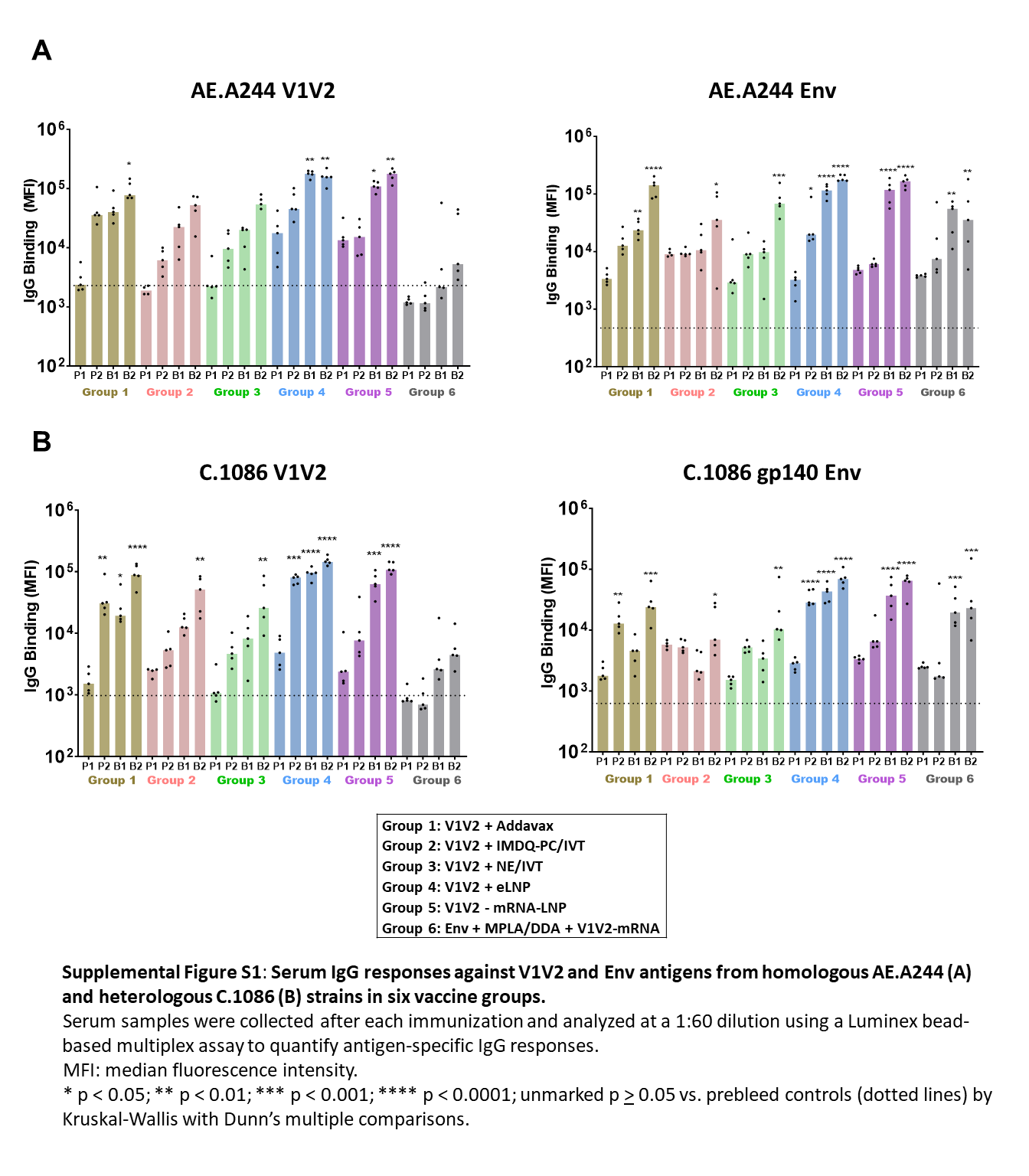

Supplement: Supplementary Figure 1 — Serum IgG responses against V1V2 and Env antigens from homologous AE.A244 (A) and heterologous C.1086 (B) strains in six vaccine groups. Serum samples were collected after each immunization and analyzed at a 1:60 dilution using a Luminex bead-based multiplex assay to quantify antigen-specific IgG responses. MFI: median fluorescence intensity. *p < 0.05; **p < 0.01; ***p < 0.001; ****p < 0.0001; unmarked p ≥ 0.05 vs. prebleed controls (dotted lines) by Kruskal-Wallis with Dunn’s multiple comparisons. [file Image1.tif]

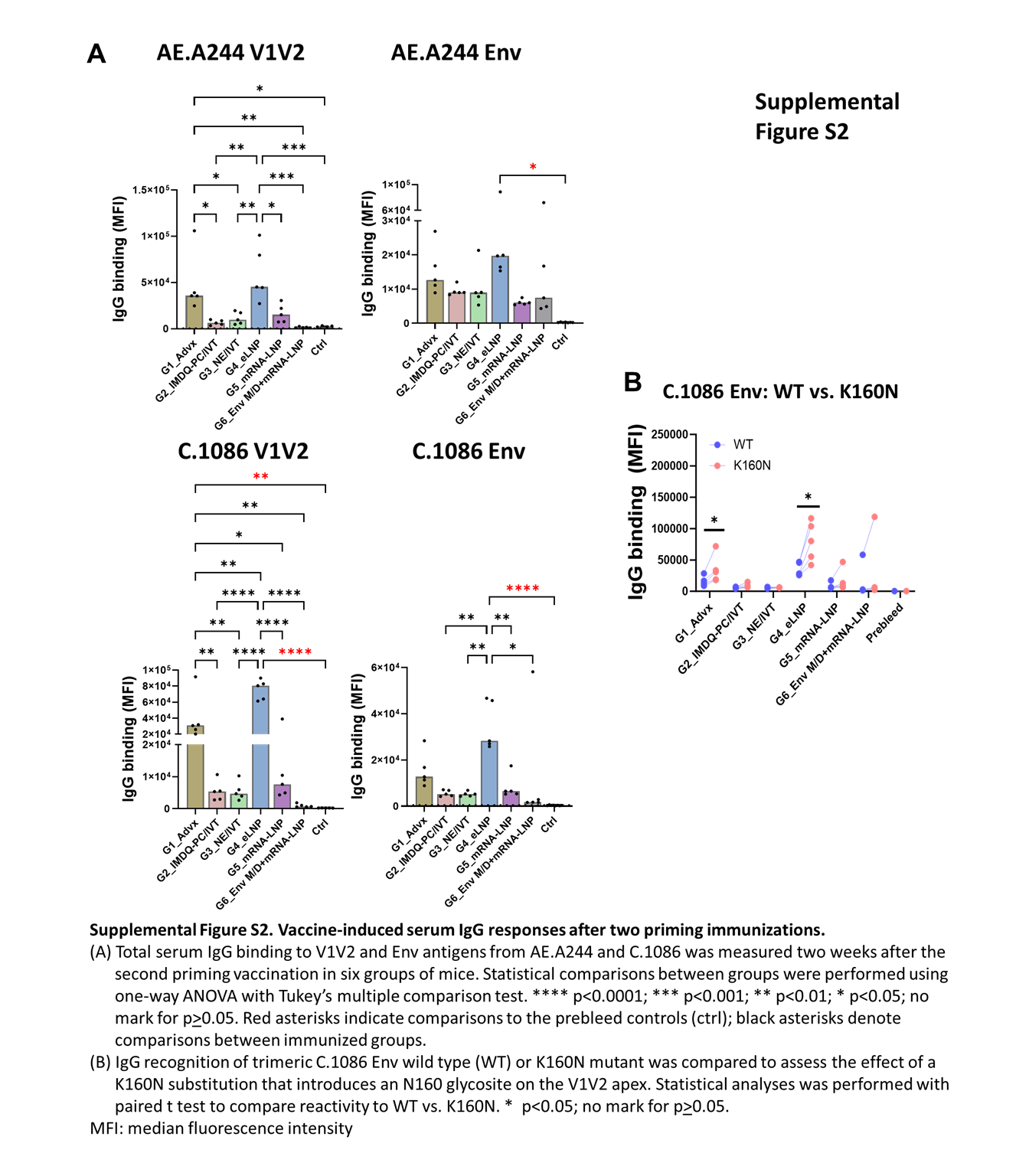

Supplement: Supplementary Figure 2 — Vaccine-induced serum IgG responses after two priming immunizations. (A) Total serum IgG binding to V1V2 and Env antigens from AE.A244 and C.1086 was measured two weeks after the second priming vaccination in six groups of mice. Statistical comparisons between groups were performed using one-way ANOVA with Tukey’s multiple comparison test. ****p<0.0001; ***p<0.001; **p<0.01; *p<0.05; no mark for p≥0.05. Red asterisks indicate comparisons to the prebleed controls (ctrl); black asterisks denote comparisons between immunized groups. (B) IgG recognition of trimeric C.1086 Env wild type (WT) or K160N mutant was compared to assess the effect of a K160N substitution that introduces an N160 glycosite on the V1V2 apex. Statistical analyses was performed with paired t test to compare reactivity to WT vs. K160N. *p<0.05; no mark for p≥0.05. MFI: median fluorescence intensity. [file Image2.tif]

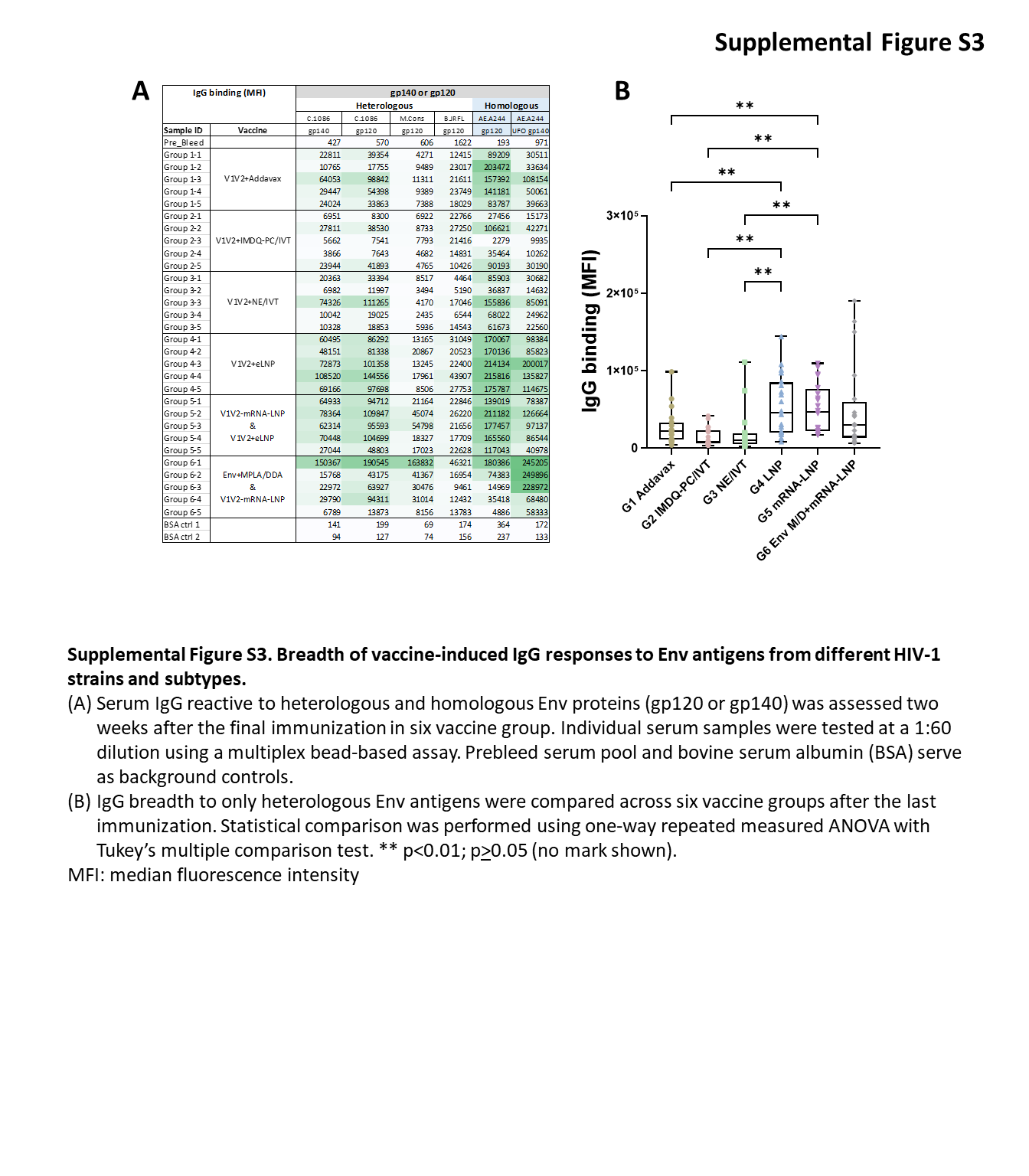

Supplement: Supplementary Figure 3 — Breadth of vaccine-induced IgG responses to Env antigens from different HIV-1 strains and subtypes. (A) Serum IgG reactive to heterologous and homologous Env proteins (gp120 or gp140) was assessed two weeks after the final immunization in six vaccine group. Individual serum samples were tested at a 1:60 dilution using a multiplex bead-based assay. Prebleed serum pool and bovine serum albumin (BSA) serve as background controls. (B) IgG breadth to only heterologous Env antigens were compared across six vaccine groups after the last immunization. Statistical comparison was performed using one-way repeated measured ANOVA with Tukey’s multiple comparison test. **p<0.01; p≥0.05 (no mark shown). MFI: median fluorescence intensity. [file Image3.tif]

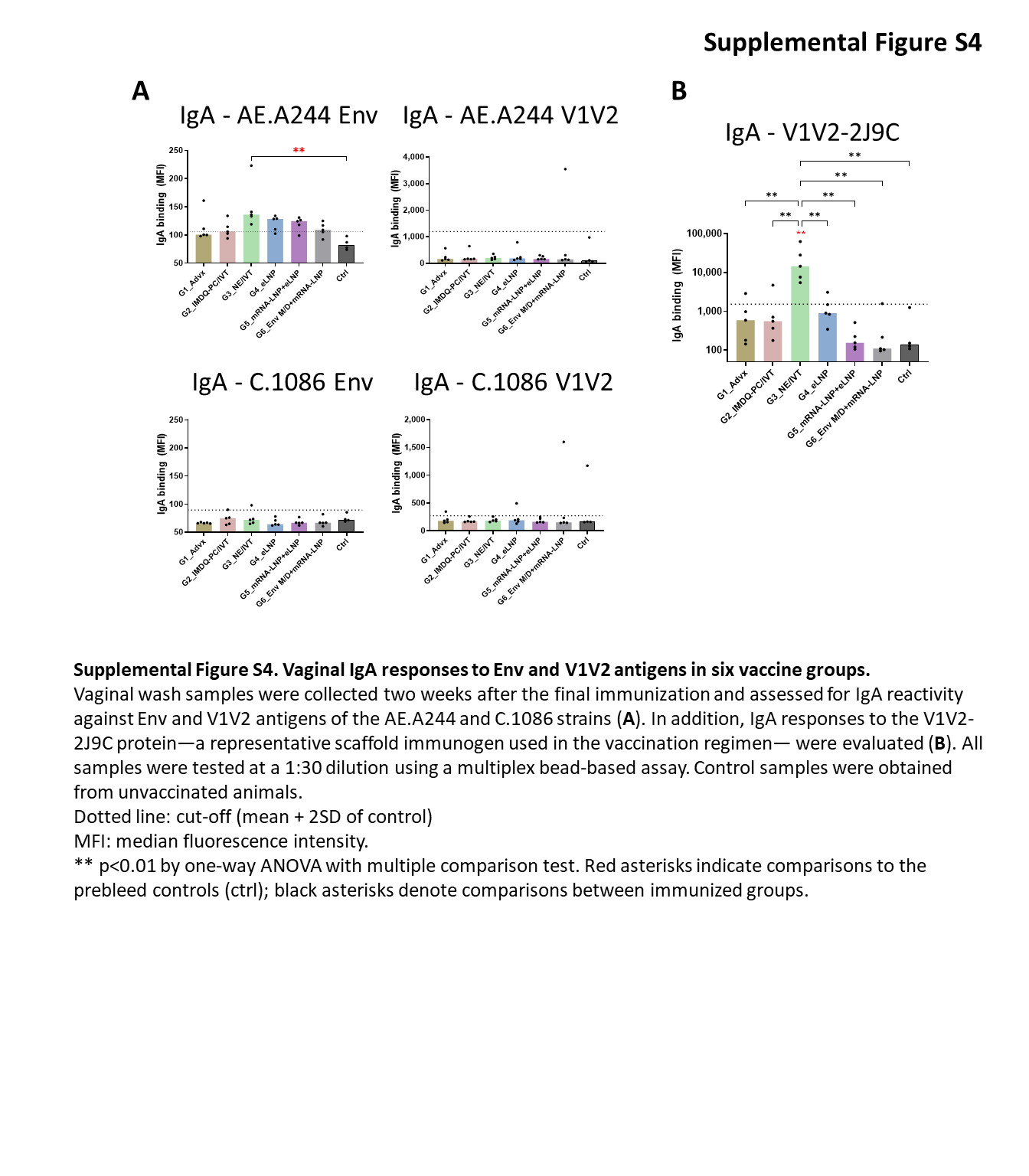

Supplement: Supplementary Figure 4 — Vaginal IgA responses to Env and V1V2 antigens in six vaccine groups. Vaginal wash samples were collected two weeks after the final immunization and assessed for IgA reactivity against Env and V1V2 antigens of the AE.A244 and C.1086 strains (A). In addition, IgA responses to the V1V2-2J9C protein—a representative scaffold immunogen used in the vaccination regimen— were evaluated (B). All samples were tested at a 1:30 dilution using a multiplex bead-based assay. Control samples were obtained from unvaccinated animals. Dotted line: cut-off (mean + 2SD of control). MFI: median fluorescence intensity. **p<0.01 by one-way ANOVA with multiple comparison test. Red asterisks indicate comparisons to the prebleed controls (ctrl); black asterisks denote comparisons between immunized groups. [file Image4.tif]

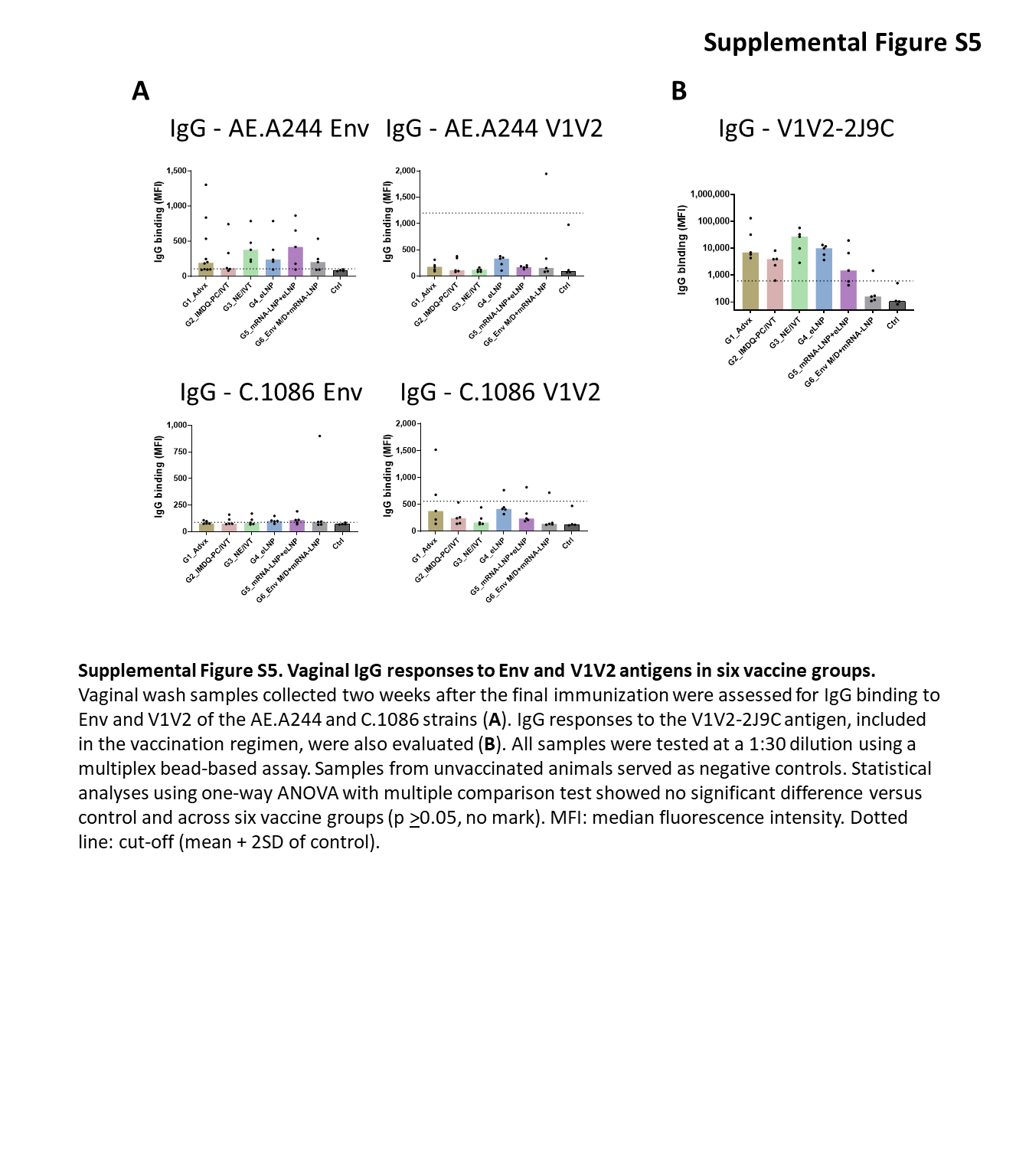

Supplement: Supplementary Figure 5 — Vaginal IgG responses to Env and V1V2 antigens in six vaccine groups. Vaginal wash samples collected two weeks after the final immunization were assessed for IgG binding to Env and V1V2 of the AE.A244 and C.1086 strains (A). IgG responses to the V1V2-2J9C antigen, included in the vaccination regimen, were also evaluated (B). All samples were tested at a 1:30 dilution using a multiplex bead-based assay. Samples from unvaccinated animals served as negative controls. Statistical analyses using one-way ANOVA with multiple comparison test showed no significant difference versus control and across six vaccine groups (p ≥0.05, no mark). MFI: median fluorescence intensity. Dotted line: cut-off (mean + 2SD of control). [file Image5.tif]

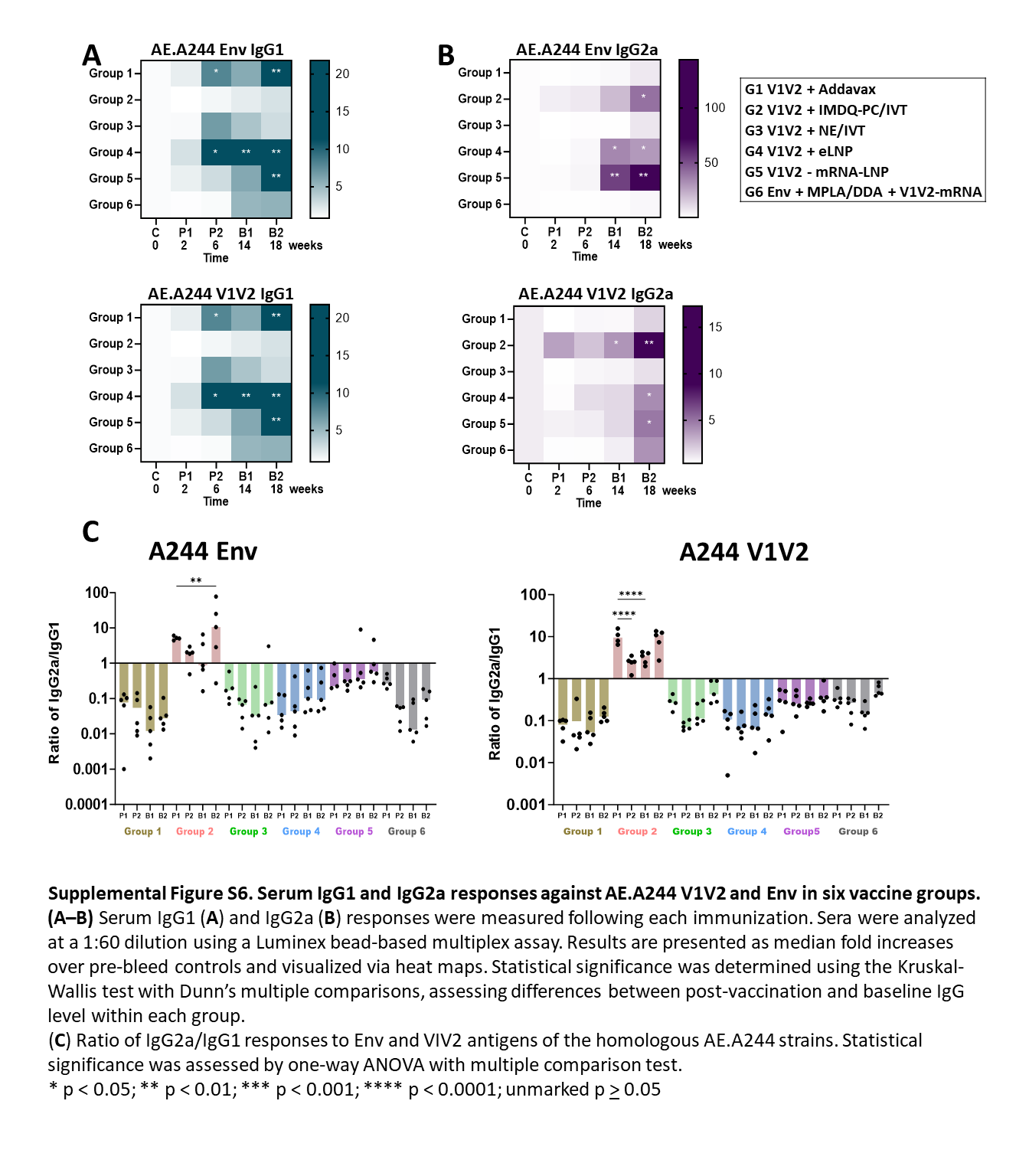

Supplement: Supplementary Figure 6 — Serum IgG1 and IgG2a responses against AE.A244 V1V2 and Env in six vaccine groups. (A–B) Serum IgG1 (A) and IgG2a (B) responses were measured following each immunization. Sera were analyzed at a 1:60 dilution using a Luminex bead-based multiplex assay. Results are presented as median fold increases over pre-bleed controls and visualized via heat maps. Statistical significance was determined using the Kruskal-Wallis test with Dunn’s multiple comparisons, assessing differences between post-vaccination and baseline IgG level within each group. (C) Ratio of IgG2a/IgG1 responses to Env and VIV2 antigens of the homologous AE.A244 strains. Statistical significance was assessed by one-way ANOVA with multiple comparison test. *p < 0.05; **p < 0.01; ***p < 0.001; ****p < 0.0001; unmarked p > 0.05. [file Image6.tif]

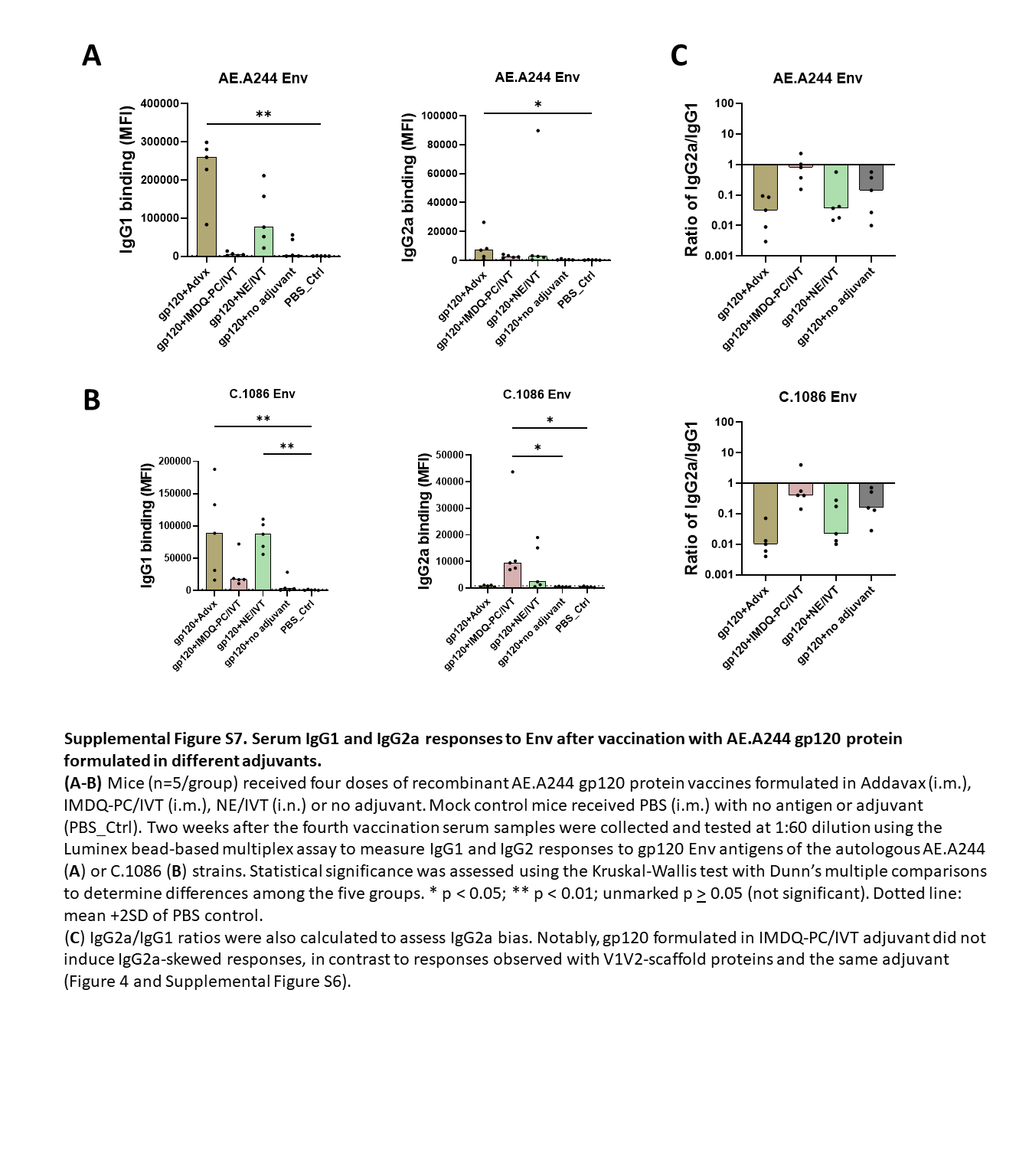

Supplement: Supplementary Figure 7 — Serum IgG1 and IgG2a responses to Env after vaccination with AE.A244 gp120 protein formulated in different adjuvants. (A, B) Mice (n=5/group) received four doses of recombinant AE.A244 gp120 protein vaccines formulated in Addavax (i.m.), IMDQ-PC/IVT (i.m.), NE/IVT (i.n.) or no adjuvant. Mock control mice received PBS (i.m.) with no antigen or adjuvant (PBS_Ctrl). Two weeks after the fourth vaccination serum samples were collected and tested at 1:60 dilution using the Luminex bead-based multiplex assay to measure IgG1 and IgG2 responses to gp120 Env antigens of the autologous AE.A244 (A) or C.1086 (B) strains. Statistical significance was assessed using the Kruskal-Wallis test with Dunn’s multiple comparisons to determine differences among the five groups. *p < 0.05; **p < 0.01; unmarked p ≥ 0.05 (not significant). Dotted line: mean +2SD of PBS control. (C) IgG2a/IgG1 ratios were also calculated to assess IgG2a bias. Notably, gp120 formulated in IMDQ-PC/IVT adjuvant did not induce IgG2a-skewed responses, in contrast to responses observed with V1V2-scaffold proteins and the same adjuvant (Figure 4, Supplementary Figure S6). [file Image7.tif]

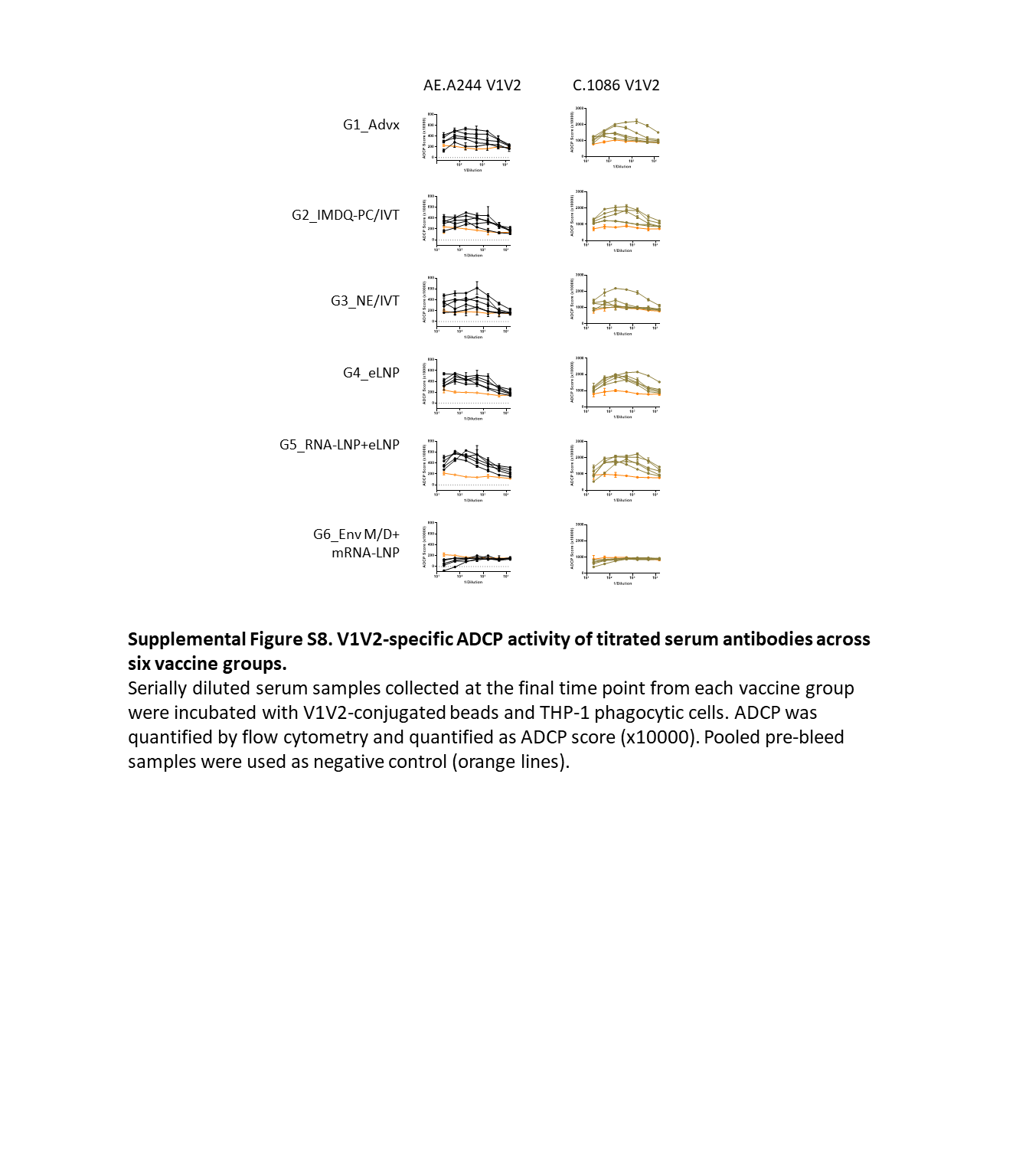

Supplement: Supplementary Figure 8 — V1V2-specific ADCP activity of titrated serum antibodies across six vaccine groups. Serially diluted serum samples collected at the final time point from each vaccine group were incubated with V1V2-conjugated beads and THP-1 phagocytic cells. ADCP was quantified by flow cytometry and quantified as ADCP score (x10000). Pooled pre-bleed samples were used as negative control (orange lines). [file Image8.tif]

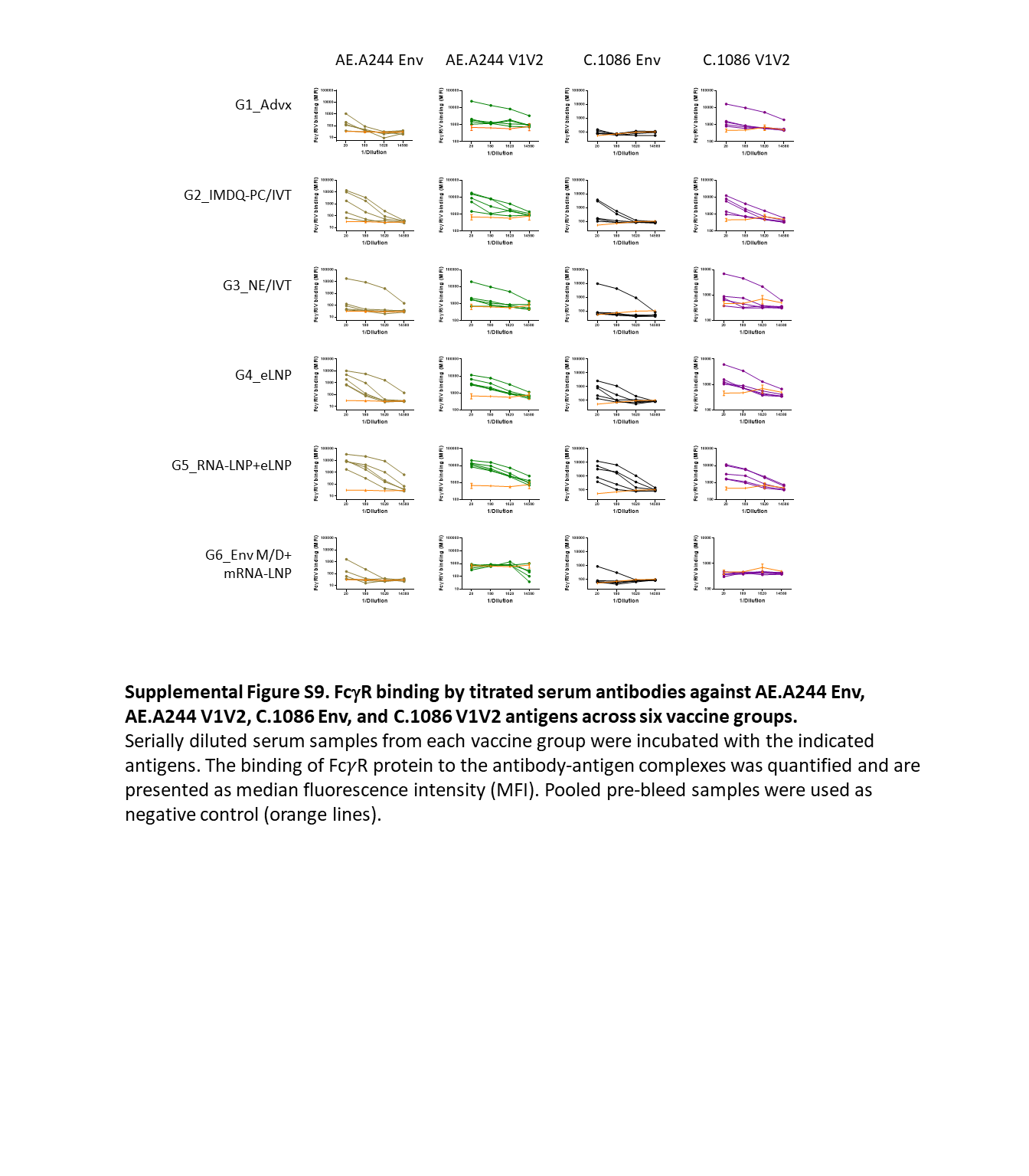

Supplement: Supplementary Figure 9 — FcγR binding by titrated serum antibodies against AE.A244 Env, AE.A244 V1V2, C.1086 Env, and C.1086 V1V2 antigens across six vaccine groups. Serially diluted serum samples from each vaccine group were incubated with the indicated antigens. The binding of FcγR protein to the antibody-antigen complexes was quantified and are presented as median fluorescence intensity (MFI). Pooled pre-bleed samples were used as negative control (orange lines). [file Image9.tif]
